# Perceptions and Sentiments About Electronic Cigarettes on Social Media Platforms: Systematic Review

**DOI:** 10.2196/13673

**Published:** 2020-01-15

**Authors:** Misol Kwon, Eunhee Park

**Affiliations:** 1 School of Nursing University at Buffalo Buffalo, NY United States

**Keywords:** electronic cigarettes, electronic nicotine delivery systems, internet, social media, review

## Abstract

**Background:**

Electronic cigarettes (e-cigarettes) have been widely promoted on the internet, and subsequently, social media has been used as an important informative platform by e-cigarette users. Beliefs and knowledge expressed on social media platforms have largely influenced e-cigarette uptake, the decision to switch from conventional smoking to e-cigarette smoking, and positive and negative connotations associated with e-cigarettes. Despite this, there is a gap in our knowledge of people’s perceptions and sentiments on e-cigarettes as depicted on social media platforms.

**Objective:**

This study aimed to (1) provide an overview of studies examining the perceptions and sentiments associated with e-cigarettes on social media platforms and online discussion forums, (2) explore people’s perceptions of e-cigarette therein, and (3) examine the methodological limitations and gaps of the included studies.

**Methods:**

Searches in major electronic databases, including PubMed, Cumulative Index of Nursing and Allied Health Literature, EMBASE, Web of Science, and Communication and Mass Media Complete, were conducted using the following search terms: “electronic cigarette,” “electronic vaporizer,” “electronic nicotine,” and “electronic nicotine delivery systems” combined with “internet,” “social media,” and “internet use.” The studies were selected if they examined participants’ perceptions and sentiments of e-cigarettes on online forums or social media platforms during the 2007-2017 period.

**Results:**

A total of 21 articles were included. A total of 20 different social media platforms and online discussion forums were identified. A real-time snapshot and characteristics of sentiments, personal experience, and perceptions toward e-cigarettes on social media platforms and online forums were identified. Common topics regarding e-cigarettes included positive and negative health effects, testimony by current users, potential risks, benefits, regulations associated with e-cigarettes, and attitude toward them as smoking cessation aids.

**Conclusions:**

Although perceptions among social media users were mixed, there were more positive sentiments expressed than negative ones. This study particularly adds to our understanding of current trends in the popularity of and attitude toward e-cigarettes among social media users. In addition, this study identified conflicting perceptions about e-cigarettes among social media users. This suggests that accurate and up-to-date information on the benefits and risks of e-cigarettes needs to be disseminated to current and potential e-cigarette users via social media platforms, which can serve as important educational channels. Future research can explore the efficacy of social media–based interventions that deliver appropriate information (eg, general facts, benefits, and risks) about e-cigarettes.

**Trial Registration:**

PROSPERO CRD42019121611; https://tinyurl.com/yfr27uxs

## Introduction

Although the prevalence of cigarette smoking has been decreasing in the last decades, electronic cigarette (e-cigarette) use, on the contrary, has been increasing dramatically [1]. E-cigarettes have been portrayed on social media platforms as a means of providing craving relief or reducing cigarette consumption for those wanting to quit [2,3]. However, recent findings state that e-cigarettes’ impact on users’ health and well-being needs to be studied in depth and with a long-term follow-up to validate such conclusions [1,4]. Considering the drastic increase in e-cigarette use and the uncertainty of its usefulness and consequences, people are turning to social media platforms for up-to-date information.

As internet use and mobile phone ownership have become a nearly ubiquitous element of people’s lives in the last decade, the internet has provided platforms where people search for information and create communities around a shared interest [5]. Social media platforms can be defined as internet-based or mobile app–based communities that facilitate the creation and exchange of user-generated content through activities that range from photo and video sharing to social networking and crowdsourcing [6]. They provide a framework for people to connect, network, build, and thrive on the Web [7]. Twitter, a free social networking service, primarily focuses on microblogging [8], where its users can communicate via short messages with a maximum of 280 characters called *tweets*. These *tweets* can be instantly transmitted to *followers* of the account via the Twitter website or mobile phone app, or email [8]. Facebook, online news sources, photography-based storytelling social networking apps (eg, Instagram), and community-style picture posting and organizing apps (eg, Pinterest) are other popular platforms, where people search and share the information [9]. Common social media platforms where smokers can share e-cigarette–related information include Twitter and Facebook.

Discussion-based social media platforms, which are often called online forums, host conversations between users who post messages. It allows asynchronous interactions through which participants can engage or observe discussions at their convenience on a topic of their interest. Reddit is an example of a collection of forums where users can share interesting links, images, and posts. JuiceDB is another example, which provides website- and app-based online forums that allow people to discuss their thoughts about e-cigarettes. In view of this, data from social media platforms can be used by public health researchers to gain insights and understand public opinion on current public health–related phenomena and inform the design of public health surveillance [10].

Social media platforms are a popular way for people to share personal experience and exchange information about health [11]. More than 70% of the population has reported using more than one social media platform, and the proportion of social media users who state difficulty living without these platforms continues to increase [12,13]. On social media platforms, people can easily share pictures, information, interests, experiences, sentiments, and opinions about health and risk-taking behaviors, including the use of e-cigarettes. Hence, the depiction of e-cigarettes on social media platforms is on the rise [9,14], and it may have contributed to the heightening of curiosity, approval, and experimentation among many routine internet users seeking reviews of the actual experience [15]. Interestingly, tobacco users are 5 times more likely to share information about e-cigarettes across social media platforms than nonusers [9]. These days, social media platforms have become a medium for both members of the medical community as well as general users in providing opportunities to voice their input about vaping devices and e-liquid products and obtain information from other users [11]. This may be related to the short supply of usage and safety guidelines on vaping devices and products for current and potential e-cigarette users and health care providers.

With limited knowledge of the public’s perceptions and sentiments toward e-cigarettes, social media platforms can act as major sources of information for researchers, policy makers, and educators. A recent scoping review provides a review on the messages presented in e-cigarette–related social media promotions and discussions in the studies published in 5 developed countries [16]. McCausland et al provided important insights on e-cigarette–related messages depending on the social media account type and revealed the most common themes as health, safety, and harms [16]. In addition, selected studies were analyzed for emotional tone, affective content, or message attitudes [16]. However, we still have a limited understanding of this phenomenon, and there is a need for a systematic review on people’s perceptions and sentiments on e-cigarettes as expressed on social media platforms and online forums. This review expands on the previous scoping review and contributes to the literature by (1) adding information on online forums based on discussions by the public, which have the potential to better understand the general population, as well as subgroups; (2) providing an understanding of people’s perceptions and sentiments, including in-depth reasons for using or not using e-cigarettes based on the synthesis of the findings; (3) adding insights using different search engines; and (4) evaluating the methodological strengths and gaps in the literature. The aims of conducting this systematic review were to (1) provide an overview of studies examining perceptions and sentiments about e-cigarettes on social media platforms and online forums, (2) explore people’s perceptions and sentiments about e-cigarettes on social media platforms and online forums, and their potential impact on public health, and (3) examine methodological limitations and gaps of the selected studies.

## Methods

### Overview

**Figure 1:**
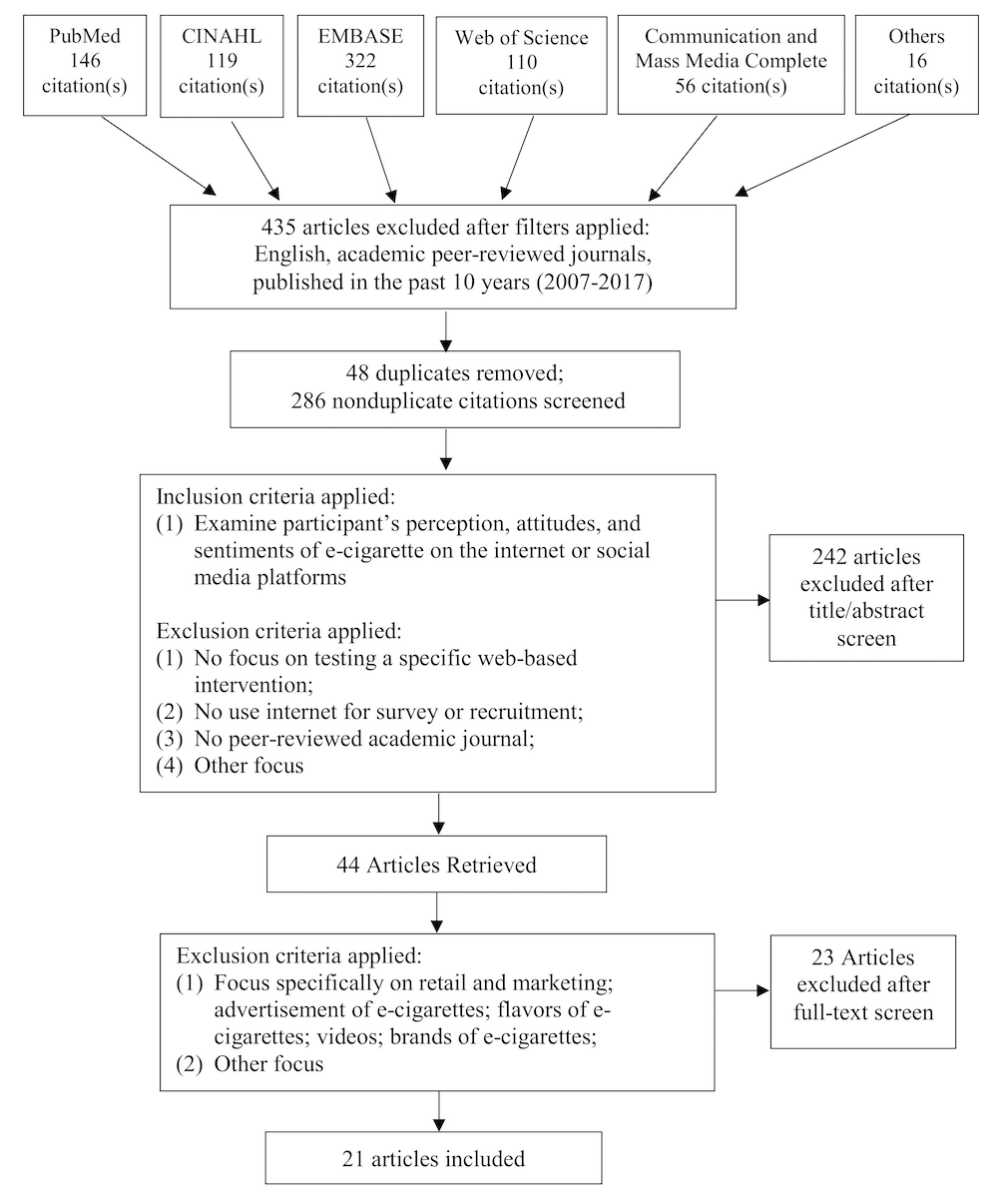
Flowchart of the literature search process. CINAHL: Cumulative Index of Nursing and Allied Health Literature.

The authors followed the guidelines of the Preferred Reporting Items for Systematic Reviews and Meta-Analyses [17]. This review is registered on PROSPERO (International Prospective Register of Systematic Reviews, CRD42019121611). Inclusion and exclusion criteria used for studies selected is shown in Figure 1.

### Search Strategy

Studies were searched from 5 major electronic databases: PubMed, the Cumulative Index of Nursing and Allied Health Literature, EMBASE, Web of Science, and Communication and Mass Media Complete. In addition, we conducted an additional search using a snowballing approach through Google Scholar. Search terms included the following keywords: e-cigarette-related terms (“electronic cigarette” OR “electronic vaporizer” OR “electronic nicotine” OR “electronic nicotine delivery systems [MeSH]”) AND social media platform-related terms (“internet [MeSH],” “social media [MeSH],” OR “internet use”). To obtain a more comprehensive and accurate search outcome, we used controlled vocabulary (ie, MeSH [Medical Subject Headings] terms). MeSH is a set list of terms that includes related search terms and are set to categorize and index articles in a systematic way. For instance, the MeSH term “electronic nicotine delivery systems” encompasses “e-cigarettes” along with other related narrow terms such as “vaping.” This was applied to the terms for social media platforms and online forums by using the MeSH terms “social media” and “internet.”

Initial search was conducted from May to July 2017, and additional search was completed in May 2019.

### Eligibility Criteria

Studies were included if:

1. They were published in peer-reviewed academic journals in the past 10 years (2007-2017).
2. They examined participants’ perceptions and sentiments of e-cigarettes on the internet or social media websites.
3. They were written in English.

Studies were excluded if:

1. They were gray literature, including dissertations, conference proceeding papers, abstracts, or editorials.
2. They were using the internet as a survey tool or for participant recruitment.
3. They were focusing specifically on specific intervention, video analysis, retail and marketing or advertisement, e-cigarettes flavors, and e-cigarettes brands.

### Data Extraction, Analysis, and Synthesis

The database searches yielded a total of 769 articles. Of these, 435 articles were excluded for not meeting the inclusion criteria (articles in English, from peer-reviewed journal articles, and published in the past 10 years), leaving 334 articles, which were then imported into a citation manager for the identification of duplicates [18]. The citation manager identified and excluded duplicates (n=48); thereafter, all nonduplicate articles (n=286) were compiled into an Excel spreadsheet. The titles and abstracts of all nonduplicate articles were then reviewed by 4 researchers (YB, MF, MK, and EP), including 2 authors (MK and EP) and 2 other researchers (YB and MF), who determined whether they met the predetermined inclusion criteria. Most articles were excluded in this first screening process if they did not focus on e-cigarettes, were not based on social media platforms or online forums, used social media platforms for survey or recruitment, and focused on methodological aspects of conducting social media data research. Disagreements were resolved through discussion when needed. This initial phase of screening further led to the exclusion of 242 articles, leaving 44 articles for review. Thereafter, the first and second authors (MK and EP) carefully read the full text of the articles (n=44) and screened them for eligibility. At this stage, 23 articles were excluded because they focused on online marketing of e-cigarettes, specific brands or flavor of e-cigarettes, and methodological aspects of conducting research using social media platforms instead of sentiments or perceptions of e-cigarettes. The same 2 authors (MK and EP) extracted data for the finally included articles (n=21) by coding them in a data display matrix. They extracted information pertaining to the following study characteristics: author, publication year, study method (study design, sampling method, data collection, and analysis), study purpose and relevant discussion, study navigation process (search queries, number of content or posts analyzed, data collection period, and social media platform), findings including the overall sentiment of discussion on e-cigarette use (categories: pro, anti, natural, mixed, and not applicable), themes of summarized message topics, and examples of health-related comments. This coded information was cross-checked by the authors.

The overall sentiments of discussion on e-cigarette use were categorized as *pro* (positive toward e-cigarettes), *anti* (negative), *neutral*, *mixed*, or *not applicable* for each study, depending on which category had the highest percentage or was most applicable. This was achieved by first identifying the percentage of each sentiment (pro, neutral, and anti) based on the quantitative findings regarding sentiments or perceptions of e-cigarettes that the individual study reported. Each study identified postings on social media platforms (ie, each tweet on Twitter) or online forums as a unit of analysis. When the data in the study revealed a higher percentage of positive sentiments about e-cigarettes (ie, portraying e-cigarettes as cool, beneficial, better, etc), they were coded as *pro*. Similarly, sentiments were coded as *anti* when the individual study reported a higher percentage of negative sentiments (ie, e-cigarette use is unhealthy, disgusting, uncool, etc). The studies reporting a higher percentage of neutral sentiments (ie, stating a general comment and asking questions about e-cigarette) were coded as *neutral*, whereas those with mixed results were categorized as *mixed* (ie, when 2 countries had different results, different results were reported at 2 time points). The studies that did not quantify any sentiments (positive, neutral, or negative) about e-cigarette use were coded as *not applicable (N/A)*.

## Results

### Description of the Included Studies

A total of 21 articles were included in the systematic review (search strategy illustrated in Figure 1). A total of 20 social media platforms and websites were used: Twitter (n=12), Reddit (n=5), 14 online forums (Electronic cigarette forum, Hookah forum, Vapor Talk, Vapors forum, UK Vapors, All About E-Cigarette, Aussie Vapors, Baby Gaga, Vaping Underground, What to Expect, Momtastic [pregnancy forum], Totally Wicked E-Liquid, Baby Centre [United Kingdom], and Baby Center [United States]), and other social media platforms, such as Instagram (n=1), Pinterest (n=1), JuiceDB (n=1), and GLOBALink (n=1). The studies that used Twitter [9,19-29] yielded 801,574 cumulative tweets. A detailed overview of the studies is presented in Multimedia Appendices 1 and 2.

### Study Design

The studies utilized various data collection methods (Multimedia Appendix 1). Most studies (n=9) utilized the social media application programming interface (API) aggregation company such as GNIP, Inc [19,25,28], Twitter API [20,21,23,29], and JuiceDB API [22], and Instagram API [30,31] for data collection, whereas others (n=4) used analytics software such as NodeXL [26] and databases such as MDigitalLife Health Ecosystem [9], MySQL [30,32], and Sysomos HeartBeat [24]. Data were collected manually for 4 studies [33-36], whereas 1 study made use of a Web crawler to retrieve data from Web servers directly [37].

The most frequently used search queries included “electronic cigarettes,” “e-cigarettes,” “ecigs,” “vaping,” and “vape.” Some articles used specific key terms, such as “e-cigarette ban,” “e-cigarette FDA,” “e-cigarette regulation,” “vapelife,” “e-juice,” “flavor,” “e-liquid,” “cloud-chasing,” “second hand vape,” and “vaping during pregnancy,” or the names of public health campaign using words such as *stillblowingsmoke* or *notblowingsmoke*, depending on the specific purpose of the study [19,26].

Although the most commonly used sampling strategy was purposive sampling (16/21, 76%) [9,20-22,24,26-29,33-37], a few studies (5/21, 24%) used stratified and random sampling methods [19,23,25,30,38]. When studies did not clearly indicate the type of sampling methods used (7/21, 33%) [21,23,25,27,37], we categorized study sampling based on the description that the study provided, and most were categorized as purposive sampling because they had specific purpose of sampling meeting their aims [39].

All studies were descriptive in design. In all, 9 of the included articles (43%) used both quantitative and qualitative approach [9,20,21,24-26,28-30]. Although some studies did not specifically mention whether they used qualitative or quantitative study design, the authors categorized each study based on the description of the study design and analysis [40]. For example, if the study used a qualitative approach, such as the thematic analysis when coding, the authors categorized it as qualitative (7/21, 33%) [19,22,27,31,33-35]. If the results were reported numerically, they were categorized as quantitative (5/21, 24%) [23,32,36-38]. Data analysis techniques included text mining and modeling [9,21,23,27,37], thematic analysis [23,33], content analysis [19-21,25,26,29,32,35,38], valence analysis [28], and image analysis [30], whereas quantitative approach included descriptive statistics (eg, to report the frequencies of data based on themes), *f* statistics, chi-square statistics, Kruskal-Wallis tests, and Dunn tests [19,20,23-26,28-32,35,38].

### Major Themes About Electronic Cigarettes on Social Media Platforms

The main themes regarding e-cigarette use on social media platforms were motivation for using e-cigarettes and concerns about the health outcomes associated with their use. There were debates about their harmfulness and safety, for example, their effectiveness in promoting smoking cessation compared with conventional cigarettes, or harmfulness because of nicotine content, presence of chemicals, and the possibility of gateway effect to conventional smoking [9,19,21,22,24,26,32,34]. In addition, other major issues related to e-cigarettes were identified, which included policy, advertisement and marketing, flavor, feelings of e-cigarette use, and use among young people. These findings indicate the wide range of information available about e-cigarettes that people share on social media platform, in addition to the topics related to e-cigarettes that people are interested in and curious about (Multimedia Appendix 2).

#### Overall Perceptions About Electronic Cigarettes

The perceptions about e-cigarette use were depicted on various social media platform sources and online forum postings (Multimedia Appendix 2 and Table 1). Overall, 47.6% (n=10) of the studies were categorized as *pro* because they indicated positive perceptions about e-cigarettes [19,20,22-24,26,27,30,32,38], 19.0% (n=4) as *neutral* [21,28,31], 4.8% (n=1) as *anti* [36], and 9.5% (n=2) as *mixed* [9,25], and the studies with no data on perceptions about e-cigarette use were coded as *N/A* (19.0%, n=4) [33,37]. These details are presented in Table 1. In addition, the examples of health-related quotations are provided in Multimedia Appendix 2.

**Table 1:** Overall sentiment of discussion on electronic cigarette (e-cigarette) use (coded as pro, anti, neutral, mixed, and not applicable).

Overall sentiment and studies (first author, year)		Details	
**Pro**			
	Allem, 2017 [19]		Provaping=92%, neutral=6%, anti=2%
	Lazard, 2016 [27]		Pro=68%, neutral=32%, anti=0%
	Ayers, 2017 [20]		Reasons for using e-cigarette: quitting combustibles (43%), social image (21%), can vape indoors (17%), flavor choices (14%), safe to use (9%), low cost (3%), and favorable order (2%)
	Zhan, 2017 [22]		Reddit: pro=60.7% opponents on e-cigarette bans, neutral=29.9%, anti=9.4% proponents on e- cigarette bans
	Kavuluru, 2016 [23]		Proponents versus others: mean positive scores (0.92 and 0.79), mean negative scores (0.01 and 0.03)
	van der Tempel, 2016 [24]		Attitude: complete sample versus industry-free sample (pro=79% versus 62%, anti=12% versus 17%, neutral=8% versus 21%); Affective content: complete sample versus industry-free sample (pro=46% versus 27%, anti=7% versus 15%)
	Chu, 2015 [32]		Pro=61.9%, anti=47.7%, neutral=8.6%
	Harris, 2014 [26]		Pro=89.2% opponents of e-cigarette regulation (antipolicy), anti=7.5% proponents of e-cigarette regulation (propolicy), neutral=3.4% unable to tell
	Lee, 2017 [38]		—^a^
	Chu, 2016 [30]		—
**Anti**			
	Hua, 2013 [36]		Anti=80.5% (negative symptoms), pro=19.3% (positive symptoms), neutral=0.02% (neutral)
**Neutral**			
	Burke-Garcia, 2017 [28]		Neutral=88%-90%, pro=6%, anti=4%-5%
	Dai, 2016 [21]		Neutral**=**19.4%, anti=17.7%, pro=10.8%
	Laestadius, 2016 [31]		Neutral: presence of social identity or vaping community (81.2%), depiction of e-cigarette=up to 62.4%; pro=48.3%
	Unger, 2016 [29]		Neutral=39.24%, pro=34.96%, anti=25.81%
**Mixed**			
	Glowacki, 2017 [9]		United States: anti=54%, pro=28%, neutral=18%; United Kingdom: pro=43%, anti=37%, neutral=19%
	Cole-Lewis, 2015 [25]		Initially, pro=71.11%, neutral=16.78%, anti=12.11%, but showed steady decline in positive sentiment from December 2013
**Not applicable**			
	Sharma, 2017 [34]		—
	Wigginton, 2017 [33]		—
	Li, 2016 [35]		—
	Chen, 2015 [37]		—

^a^Cumulative percentage not provided.

#### Reasons and Motivations for Using Electronic Cigarettes

The main reasons for the popularity of e-cigarettes were identified as the benefits associated with their use, with e-cigarettes not only being used as smoking cessation devices but also being the cheaper and healthier alternatives to conventional cigarettes because of their content and environment-friendly nature [31]. The proponents viewed c-cigarettes as a harm reduction and smoking cessation aid with favorable features, such as the smoke-free vaping source with flavors [23]. In addition, e-cigarettes were depicted as more economical and efficient nicotine delivery systems than conventional smoking [24].

Interestingly, the major reasons for e-cigarette use in tweets changed from 2012 to 2015 [20]. In the past (2012), the most prevalent reasons for using e-cigarettes were quitting combustibles (43%), caring for social image (21%), and being able to use them indoors (17%). Minor reasons included choices of flavor (14%), safety relative to combustibles (9%), low cost (3%), and favorable odors (2%) [20]. However, 3 years later, in 2015, a significant decrease was seen for the reasons quitting combustibles and being able to use indoors, and the most prevalent reasons for using e-cigarettes changed to social image (37%, 95% CI 32-43), quitting combustibles (29%), and capability to smoke indoors (12%) on Twitter [20].

For people with mental illness, the motivation for using e-cigarettes was quitting smoking [34]. Particularly, with smoking cessation from other nicotine replacements with concurrent use of psychiatric medicine being unsuccessful, e-cigarettes began to be viewed as a healthier alternative. In addition, the switch to e-cigarette was made with the intention to relieve symptoms as either a self-medication or replacement of psychiatric drugs and to gain a sense of freedom, control, and social connectedness [34].

On the discussion regarding safety concerns of e-cigarette use during pregnancy, posts emphasized the dangers of abruptly stopping nicotine use (eg, physical and psychological harm of nicotine withdrawal for the mother and baby) [33]. Overall, e-cigarette use during pregnancy was viewed as a harm reduction approach, and vaping was seen as a safer alternative rather than focusing on the harmful effects of nicotine [33]. Nevertheless, some mentioned the unknown risks associated with vaping or that there was limited current scientific evidence to support vaping during pregnancy [33].

#### Smoking Aid, Cessation Method, and Harm Reduction

Discussion about e-cigarette use mainly centered on their use as a cessation aid and as a healthier alternative to combustors [9,20,26,29,31,33,37]. The proponents of e-cigarette use were more likely to tweet on the aspects of harm reduction of e-cigarettes [33], smoke-free aspects, and smoking cessation effect than other users [23], and this was also indicated in the tweets related to secondhand vaping [29]. Notably, only 6.3% of e-cigarette–related tweets were about e-cigarette use for smoking cessation [25].

Data from Vapor Talk and Reddit demonstrated extensive discussion on e-cigarette use for quitting conventional smoking [37]. E-cigarette users experienced less psychological difficulties in quitting smoking compared with combustible cigarette users [37]. The corporate users (vendors, brands, and representatives of tobacco companies or retailors) and the general e-cigarette users had positive views regarding the cessation effect on Instagram as shown in the 23.5% of the total posts [31]. In particular, Instagram posts (16.5%) depicted e-cigarettes to be healthier than tobacco products and more environment-friendly (1.2%) [31]. Similarly, the UK physicians’ tweets placed emphasis on promotion of e-cigarettes (18%) because these could serve as an effective aid for smoking cessation, followed by the discussion on general practitioner to encourage patients who smoke conventional cigarettes to switch to e-cigarettes (13%) [9].

#### Limitations and Barriers to Using Electronic Cigarette

One of the major barriers identified was a concern regarding the possibilities of e-cigarettes serving as a gateway to conventional cigarette smoking among nonusers, especially with respect to the young population, and its effect on short- and long-term health outcomes [9,19,21,36,37]. People with mental illness uniquely reported limitations to use e-cigarettes such as health concerns for replacing psychiatric medicines, drug interactions, practical difficulties, and costs, whereas the general population indicated concerns involving nicotine addiction, health effects, and e-cigarettes being an unsatisfactory substitute for tobacco products [34].

#### Health Effects and Safety

The effects on health outcomes was one of the major themes among the users of the online discussion forums and Twitter [9,19,29,31,33,36,37]. In all, 13% of tweets were related to health effects and safety issues [25]. Of the reported physical health symptoms across 10 organ systems (eg, respiratory and neurological) and 2 anatomical regions (chest and mouth/throat) among the e-cigarette users, more negative symptoms (82.2%) such as insomnia and dry lips and tongue were reported compared with the positive symptoms (17.8%) such as controlled appetite and eliminated snoring on the Electronic Cigarettes Forum [36]. Subsequently, among the groups of US and UK physicians, about 15% of tweets were regarding the effects on health outcomes such as the effect of flavoring chemicals on the lungs [9]. The effects of e-cigarettes on complications for breast reconstruction surgery were also discussed among the UK physicians [9].

Although health effects were a major concern for e-cigarette use and were seen as a barrier, mixed opinions and discussions about the ingredients of e-cigarettes were displayed. On Twitter, opponents claimed that some ingredients in e-cigarettes were carcinogenic, focusing especially with the increased use among teens (propolicy, 2.8%). However, the proponents argued that research had shown that e-cigarettes only contain nicotine and water and, hence, presented no danger with the secondhand vapor (antipolicy, 31.9%) [26]. The proponents’ main claim was that e-cigarettes may not be more harmful than conventional cigarettes [26]. Health-related tweets related to secondhand vaping were mostly anti–e-cigarettes (70%) with mentions of short- and long-term health effects of exposure to e-cigarette aerosol, such as headache, eye irritation, nausea, and lung disease [29]. Moreover, women who smoke during pregnancy described quitting nicotine as more harmful to their body and baby than cutting down the dose or frequency of smoking, indicating that vaping can be used to not only reduce harm but also replace smoking as a safer and healthier alternative during pregnancy [33].

The pros and cons of e-cigarettes compared with those of conventional cigarettes were a major discussion theme among the UK physicians with 19% of tweets [9], whereas 12% of tweets were regarding Public Health England’s recommendation that e-cigarettes were safer than the traditional forms of tobacco use [9]. Interestingly, there were no negative posts on Instagram and their posts (16.5%) that presented e-cigarettes as healthier alternative to conventional tobacco products and as environment-friendly (1.2%) [31].

### Other Issues About Electronic Cigarettes on Social Media

In addition to the major discussions on the effects of e-cigarettes on smoking cessation and their potential health concerns, there were extended discussions on the policy and regulation, flavor and techniques, feelings, symptoms, features, marketing, and youth e-cigarette use [9,19,22-26,35-37] (Multimedia Appendix 2).

#### Policy and Regulation

The debate on e-cigarette ban regulations was a commonly discussed topic [19,22,25-27]. One of the main platforms for the policy and regulation discussion was Twitter with 20.2% of tweets associated with policy and government-related issues [25]; the major proportion of those on antipolicy side discussed about the safety (52.4%) and lies/propaganda (32.8%), whereas those on the propolicy side focused more on regulation (6.4), science (2.8%), and safety (2%) [26]. In an attempt to understand the public’s initial reactions to the Food and Drug Administration’s new rule that extends their regulatory authority to include all tobacco products, including e-cigarettes, cigars, pipe tobacco, and hookah in May 2016, the study revealed many expressed comments, opinions, words, and phrases commonly associated with advocating for vaping and support for the use of e-cigarettes [27].

The frequent themes on Twitter campaigns using hashtags to express policy-related opinions included tax, individual freedom and rights, simple opposition, and call to action [22]. Most tweets generated for the California campaign were found to be mostly from outside of California [19]. Another study analyzed the responses to the campaign by the Chicago Department of Public Health [26] and presented with a considerably higher number of antipolicy tweets than propolicy tweets, which was contrary to the intention of the campaign. Higher percentage of propolicy tweets were from the Chicago residents, whereas antipolicy tweets were from outside residents. In addition, people wanted to use safer products compared with conventional tobacco products and expressed concerns about propaganda/lies spread by the health department or other government agencies (antipolicy, 32.8%) [26]. This trend was similar on Reddit, which showed 60.7% as opponents of e-cigarette bans and only 9.4% being the proponents [22].

#### Flavor and Technique

Flavor was identified as one of the main reasons why people used e-cigarettes and also as the common interest among e-cigarette users [22,26]. Specifically, Reddit and JuiceDB showed rich discussions about flavors [22,23], and 9.7% among 1800 Instagram and Pinterest images conveyed information about popular and new juice or flavors, including ideas for creating novel flavors [38]. According to Cole-Lewis et al, about 4.5% of tweets were about flavors [25]. Interestingly, proponents were 15% more likely to tweet about flavors than other users in 2013 and 20 times more likely to tweet in 2015 [23].

Zhan et al identified flavors that were most favored among the e-cigarette users, such as fruits, cream, tobacco, menthol, beverages, sweet, seasonings, nuts, rich, spiced, cool, nutty, and coffee discussed on Reddit and JuiceDB [22]. In addition, there were topics in the Vapor Forum regarding the techniques involved in using vapor products (ie, how to get a good taste, knowing different characteristics of the juices) [37]. There were mixed opinions about flavors on Twitter [26]. Although 0.3% tweets supported the idea that sweet flavors were for kids (propolicy, 0.3%), many opposed the notion of advocating smoking to children and that adults also enjoy flavors (antipolicy, 3.7%) [26].

Overall, half of the social tweets on secondhand vaping were pro–e-cigarettes (57%), which included video links of vape performance and smoke tricks [29]. Among Instagram and Pinterest, 7.8% of images were those of performing vape tricks [38].

#### Feelings and Symptoms

Symptoms and feelings related to e-cigarette use were identified [22,35-37]. In total, 405 different symptoms related to e-cigarettes were reported and discussed, of which 318 were negative and 69 were positive [36]. Symptoms related to throat and mouth were most commonly reported [22,37]. There were different views about these symptoms, as many users enjoyed the feeling of slight throat hit, which is similar to that experienced with conventional cigarette smoking [22]; however, these symptoms were viewed as problematic experiences among users [37]. Negative symptoms were perceived as *persistent*, *worsened, or increasing*, whereas positive symptoms were *decreased, improved, or eliminated* (p. 4) [36]. Anti–e-cigarette tweets among the secondhand vape posts mentioned symptoms of headache, eye irritation, nausea, and lung disease [29].

#### Marketing and Promotion

Current e-cigarette marketing strategies and different kinds of promotion were identified [19,22,24,37]. Twitter was identified as the major source of advertisement and promotion among people because 26.3% of tweets were identified as being associated with marketing, advertisement, and promotion-related content, which was the single largest category [25]. People shared messages on specific products, coupons, vape shops for e-cigarettes, sale information, and small business on Twitter [19]. There were postings about production promotion and recommendations in the form of user review on JuiceDB and individual trades and vendor promotions on Reddit [22]. Furthermore, existing patterns of a large secondhand e-cigarette trading market, including sales from vendors to users and trades among site users was revealed [22]. In addition, vendors and end users were actively posting about specific products and sale information on e-cigarettes on the Vapor Talk and Hookah Forum [37] as well as Instagram and Pinterest [30,38].

#### Electronic Cigarette Use Among Youth

The likelihood of e-cigarette use among teenagers was another important theme [9,19,25]. The most common topic tweeted by the US physicians involved concerns about e-cigarette use among teens and the potential of tobacco addiction with the continual use of e-cigarettes among youth (21%) [9]. Similarly, organic-against tweets (17.7%) also prompted e-cigarette prevention for the general public and youth with educational information about harms associated with e-cigarettes [21].

However, although the most common topic among tweets by the US physicians was related to the dangerous rise in the use of e-cigarette among teens that displayed negative sentiment toward e-cigarette, tweets by the UK physicians had no mention of danger among youth [9]. The US physicians were also concerned that advertisement effort was aimed at teenagers and supported the notion of raising the required age for purchasing e-cigarettes [9]. Youth e-cigarette use was also a concern in another study, particularly regarding the tobacco companies’ marketing strategies among the anti–e-cigarette tweets [19], which is consistent with the fact that 4.2% of tweets were on issues regarding e-cigarette use by underage users [25].

### Methodological Evaluation

Overall, most studies included in our review were satisfactory for methodological evaluation criteria suggested by the Agency for Healthcare Research and Quality (Table 2). However, a few methodological issues have been identified (Table 2). A few studies needed to provide clearer research questions, although their studies were exploratory in nature [31,33,38]. Most studies provided thorough descriptions of methodology, such as search tools, selection methods, search terms used, and capture period, along with the rationale for data collection procedures and analysis. Most studies used purposive sampling, whereas a few studies used random sampling. Most studies did not have problems with data analysis and results reported, although more detailed descriptions about the analytic methods may have been helpful. It is because some of the analytic techniques and software used for data analysis on social media platforms were relatively new to the readers, given that social media–based research is relatively an emerging area. In addition, procedures to ensure reliability of coding (eg, double-checking by multiple coders) may need to be included in the methods [36]. Moreover, some studies lacked the clear explanation of limitations of their studies, which would be critical for the readers to consider while interpreting the findings [26,33], and more in-depth discussions could have been provided on their findings [23]. Furthermore, it may be an issue related to the journal requirement, but a few studies did not provide information on funding source of their studies [21,26,31].

**Table 2:** Methodological evaluation.

First author, year	Domains					
	Study question^a^	Data collection^b^	Data analysis^c^	Results^d^	Discussion^e^	Funding or sponsorship^f^
Allem, 2017 [19]	DCA^g^	DCA	DCA	DCA	DCA	DCA
Ayers, 2017 [20]	DCA	DCA	DCA	DCA	DCA	DCA
Burke-Garcia, 2017 [28]	DCA	DCA	DCA	DCA	DCA	DCA
Chu, 2017 [30]	DCA	DCA	DCA	DCA	DCA	DCA
Glowacki, 2017 [9]	DCA	DCA	DCA	DCA	DCA	DCA
Lee, 2017 [28]	DPA^h^	DCA	DCA	DCA	DCA	DCA
Sharma, 2017 [34]	DCA	DCA	DCA	DCA	DCA	DCA
Wigginton, 2017 [33]	DPA	DCA	DCA	DCA	DPA	DPA
Zhan, 2017 [22]	DCA	DCA	DCA	DCA	DCA	DCA
Dai, 2016 [21]	DCA	DCA	DCA	DCA	DCA	DNA^i^
Laestadius, 2016 [31]	DPA	DCA	DCA	DCA	DCA	DNA
Lazard, 2016 [27]	DCA	DCA	DCA	DCA	DCA	DNA
Li, 2016 [35]	DCA	DCA	DCA	DCA	DCA	DCA
Kavuluru, 2016 [23]	DCA	DCA	DCA	DCA	DPA	DCA
Unger, 2016 [29]	DCA	DCA	DCA	DCA	DCA	DCA
van der Tempel, 2016 [24]	DCA	DCA	DCA	DCA	DCA	DCA
Chen, 2015 [37]	DCA	DCA	DCA	DCA	DCA	DCA
Chu, 2015 [32]	DCA	DCA	DCA	DCA	DCA	DCA
Cole-Lewis, 2015 [25]	DCA	DCA	DCA	DCA	DCA	DCA
Harris, 2014 [26]	DCA	DCA	DCA	DCA	DPA	DNA
Hua, 2013 [36]	DCA	DCA	DPA	DCA	DCA	DCA

^a^Study question: Was the purpose of the study clear and focused?

^b^Data collection: Was the data collection adequately described (eg, search tool, selection manual, search terms, and capture period)?

^c^Data analysis: Was the description of the data analysis clearly described (eg, coding process, analytic techniques, classification, and statistical tests)?

^d^Results: Were the outcomes specified (eg, domains or measurement of outcomes)?

^e^Discussion: Were conclusions supported by results, with limitations taken into consideration?

^f^Funding or sponsorship: Was the type and sources of support for study mentioned?

^g^DCA: domain completely addressed.

^h^DPA: domain partially addressed.

^i^DNA: domain not addressed.

## Discussion

### Summary of Findings

Our findings enable us to gain insights regarding people’s experiences with e-cigarettes through the lens of social media platforms and discussions on online forums. Popular social media platforms such as Facebook, Instagram, and Twitter have the ability to quickly spread individual stances and opinions. They have the potential to attract the attention of daily users of social media and both indirectly and directly influence public health and global issues [19]. Overall, there was a higher volume of tweets and discussion threads for pro–e-cigarettes than anti–e-cigarettes. This finding is consistent with a previous study [16]. Positive perceptions relevant to the health effects were also seen when comparing e-cigarettes as a better alternative to conventional cigarettes. This is consistent with previous studies on general users where the majority believed that e-cigarettes were a safer alternative to conventional cigarettes and acted as an effective smoking cessation aid [41-44]. The negative perceptions mainly arose from topics such as the potential health effects of e-cigarettes, the possible gateway effect to conventional cigarettes, and the risk for addiction.

One of the issues related to e-cigarette use appearing on social media platforms and discussions on online forums included content targeting youth social media users. With the increasing number of youth being exposed to e-cigarettes on popular websites and Web-based sources [45,46], social media use can potentially contribute to the perceptions and interests of smoking among this population [47]. The role of government, policy, and propaganda appeared as another major theme. One study illustrated the power and reach of social media by suggesting how information can be easily disseminated in a short period and how even a state campaign can influence people all around the nation [19]. Furthermore, social media platforms, particularly Twitter, can be used by e-cigarette proponents, including tobacco companies and related business owners, for defending their positions [26].

The differences in perceptions on social media platforms across countries were also noted. For example, there was a difference between the UK and US physicians’ attitudes toward e-cigarettes, in that the US tweets emphasized more on the dangers of its use among youth, whereas the UK tweets focused on the potentiality of e-cigarettes to be used as the smoking cessation aid [9]. When tweets among several countries were analyzed, the United Kingdom showed the highest rate of pro–e-cigarette tweets, whereas Hungary showed the highest rate of anti–e-cigarette tweets [21]. With discussion threads, Switzerland and Canada showed more positive sentiment scores for e-cigarette topics than thread posts by the users of the United States, Australia, the United Kingdom, Ireland, Colombia, Japan, Malaysia, and Pakistan [32].

Furthermore, social media platforms reflected upon the perspectives of some of the population subsets through their e-community such as the physician groups and people with mental health issues [9,24,34]. Motivation for people with mental illness to vape included self-medication and quitting smoking, feeling of self-control, and role for hobby and social connectedness, whereas barriers to vaping included e-cigarettes being considered a low-grade substitute for cigarettes and medicine, risk of addiction, difficulties in using, and cost [34]. This finding is inconsistent with a study on a national sample of US adults where reasons for the use of e-cigarettes among those with mental health conditions were *just because*, quitting smoking, safer mode compared with conventional cigarettes, ease of use, and cost [48].

Contradictory findings were noted with respect to the users of social media platforms, although only a few studies reported on characteristics and proportions of industrial users. One study identified the proportion of users from industry on social media platforms [19]. This study used social media platforms for a public health campaign, and almost half of the total users were industrial users [19]. Another study found strategies of tobacco companies, such as using popular hashtags to increase retweets and using specific hashtags such as #quitsmoking to purposefully reach tobacco users interested in quitting [24]. Most Twitter users were identified as everyday users, with tobacco companies and retailors representing only 7.77% and 1.97%, respectively, in another study [25]. In many cases, e-cigarette companies were targeting young people while promoting their events and popular venues largely via social media platforms, and policy may need to be put in place to reduce advertisements on popular social media sites [49].

### Limitations

There are certain limitations to this review. Although we used search strategies and techniques to systematically find the articles from multiple search engines, there remains a possibility of some articles being missed. There can be potential errors in terms of incorrect categorization or elimination of relevant findings that may have contributed to the perceptions and sentiments of e-cigarettes on social media platforms despite multiple coders independently coding articles and analyzing the themes. In addition, we did not specifically include terms such as *perceptions* or *sentiments*, as we did not want to miss articles that had not used these terms in the title, abstract, or keywords by narrowing the search results with those search terms; for example, some articles explored e-cigarette sentiments or perceptions on social media platforms, but they did not use the term *sentiments* or *perceptions* in their titles, abstracts, or keywords [19-24,26-29,33,36-38]. With this search strategy, we had to screen more articles in the initial screening phase, but it yielded a broader pool of articles and lowered the chances of missing relevant articles.

### Recommendations for Future Studies

Overall, social media platforms offer benefits in research by serving as data sources for researchers and health care professionals, making it possible to collect and access valuable information regarding perceptions and sentiments of people on social media platforms and online forums. However, owing to the anonymous nature of social media users, only a few studies revealed demographic information about the users [19,23-25]. As a result, we have limited knowledge on how perceptions and sentiments vary depending on subgroups of population. Thus, future studies may need to explore how perceptions and sentiments differ based on the user characteristics, such as age, gender, race/ethnicity, and socioeconomic status. In addition, future studies can benefit by including detailed descriptions of procedures used to ensure reliability of their coding and analytic methods for the readers that may be relatively new to the concept of social media data and research.

### Conclusions

This study identifies overall trends of research regarding people’s perceptions on e-cigarettes on social media platforms and online forums. People’s perceptions and sentiments about e-cigarette use on social media platforms and online forums were more positive than negative. Positive sentiments about e-cigarettes dramatically increased on social media platforms [25], which contradicted the results of the Tobacco Products and Risk Perceptions survey in the same period where there was an increase in negative perceptions among the general public [50]. This may be related to the fact that social media platforms and online forums are being more frequently used by e-cigarette users and those who are interested in potential use or marketing. With the increasing popularity of social media use, it is possible that individuals who regard e-cigarette use as a salient social norm and helpful cessation device may post and comment and build e-communities about e-cigarettes. In addition, the positive views on social media platforms may be related to the steep increase in the use of e-cigarette among adolescents and young adults, who are more frequent social media users. Given the findings of this study, social media platforms can be important channels for intervention delivery. Web or app-based health interventions that deliver appropriate information about the harms and benefits of e-cigarette and latest research updates on new vaping devices can prove to be beneficial.

## Appendix

Multimedia Appendix 1 Overview of included articles.

Multimedia Appendix 2 Sentiments and themes identified.

## Abbreviations

API: application programming interface
e-cigarette: electronic cigarette
MeSH: Medical Subject Headings

## References

[1] Jamal A, Phillips E, Gentzke AS, Homa DM, Babb SD, King BA, Neff LJ (2018). Current cigarette smoking among adults - United States, 2016. MMWR Morb Mortal Wkly Rep.

[2] Cahn Z, Siegel M (2011). Electronic cigarettes as a harm reduction strategy for tobacco control: a step forward or a repeat of past mistakes?. J Public Health Policy.

[3] Farsalinos KE, Romagna G, Tsiapras D, Kyrzopoulos S, Voudris V (2014). Characteristics, perceived side effects and benefits of electronic cigarette use: a worldwide survey of more than 19,000 consumers. Int J Environ Res Public Health.

[4] (2016). E-cigarettes.SurgeonGeneral.

[5] Park E, Kwon M (2018). Health-related internet use by children and adolescents: systematic review. J Med Internet Res.

[6] Kaplan AM, Haenlein M (2010). Users of the world, unite! The challenges and opportunities of social media. Bus Horiz.

[7] Helmond A (2015). The platformization of the web: making web data platform ready. Soc Media Soc.

[8] Prochaska JJ, Pechmann C, Kim R, Leonhardt JM (2012). Twitter=quitter? An analysis of Twitter quit smoking social networks. Tob Control.

[9] Emery SL, Vera L, Huang J, Szczypka G (2014). Wanna know about vaping? Patterns of message exposure, seeking and sharing information about e-cigarettes across media platforms. Tob Control.

[10] Allem J, Ferrara E, Uppu SP, Cruz TB, Unger JB (2017). E-cigarette surveillance with social media data: social bots, emerging topics, and trends. JMIR Public Health Surveill.

[11] Glowacki EM, Lazard AJ, Wilcox GB (2017). E-cigarette topics shared by medical professionals: a comparison of tweets from the United States and United Kingdom. Cyberpsychol Behav Soc Netw.

[12] Anderson M, Jiang J Pew Research Center.

[13] Anderson M, Smith A (2018). Pew Research Center.

[14] Pearson JL, Richardson A, Niaura RS, Vallone DM, Abrams DB (2012). e-Cigarette awareness, use, and harm perceptions in US adults. Am J Public Health.

[15] Duke JC, Allen JA, Eggers ME, Nonnemaker J, Farrelly MC (2016). Exploring differences in youth perceptions of the effectiveness of electronic cigarette television advertisements. Nicotine Tob Res.

[16] McCausland K, Maycock B, Leaver T, Jancey J (2019). The messages presented in electronic cigarette-related social media promotions and discussion: scoping review. J Med Internet Res.

[17] Moher D, Liberati A, Tetzlaff J, Altman DG, PRISMA Group (2009). Preferred reporting items for systematic reviews and meta-analyses: the PRISMA statement. PLoS Med.

[18] EndNote.

[19] Allem J, Escobedo P, Chu K, Soto DW, Cruz TB, Unger JB (2017). Campaigns and counter campaigns: reactions on Twitter to e-cigarette education. Tob Control.

[20] Ayers JW, Leas EC, Allem J, Benton A, Dredze M, Althouse BM, Cruz TB, Unger JB (2017). Why do people use electronic nicotine delivery systems (electronic cigarettes)? A content analysis of Twitter, 2012-2015. PLoS One.

[21] Dai H, Hao J (2017). Mining social media data for opinion polarities about electronic cigarettes. Tob Control.

[22] Zhan Y, Liu R, Li Q, Leischow SJ, Zeng DD (2017). Identifying topics for e-cigarette user-generated contents: a case study from multiple social media platforms. J Med Internet Res.

[23] Kavuluru R, Sabbir AK (2016). Toward automated e-cigarette surveillance: spotting e-cigarette proponents on Twitter. J Biomed Inform.

[24] van der Tempel J, Noormohamed A, Schwartz R, Norman C, Malas M, Zawertailo L (2016). Vape, quit, tweet? Electronic cigarettes and smoking cessation on Twitter. Int J Public Health.

[25] Cole-Lewis H, Pugatch J, Sanders A, Varghese A, Posada S, Yun C, Schwarz M, Augustson E (2015). Social listening: a content analysis of e-cigarette discussions on twitter. J Med Internet Res.

[26] Harris JK, Moreland-Russell S, Choucair B, Mansour R, Staub M, Simmons K (2014). Tweeting for and against public health policy: response to the Chicago Department of Public Health's electronic cigarette Twitter campaign. J Med Internet Res.

[27] Lazard AJ, Wilcox GB, Tuttle HM, Glowacki EM, Pikowski J (2017). Public reactions to e-cigarette regulations on Twitter: a text mining analysis. Tob Control.

[28] Burke-Garcia A, Stanton CA (2017). A tale of two tools: Reliability and feasibility of social media measurement tools examining e-cigarette twitter mentions. Inform Med Unlock.

[29] Unger JB, Escobedo P, Allem J, Soto DW, Chu K, Cruz T (2016). Perceptions of secondhand e-cigarette aerosol among Twitter users. Tob Regul Sci.

[30] Chu K, Allem J, Cruz TB, Unger JB (2016). Vaping on Instagram: cloud chasing, hand checks and product placement. Tob Control.

[31] Laestadius LI, Wahl MM, Cho YI (2016). #Vapelife: An exploratory study of electronic cigarette use and promotion on Instagram. Subst Use Misuse.

[32] Chu K, Valente TW (2015). How different countries addressed the sudden growth of e-cigarettes in an online tobacco control community. BMJ Open.

[33] Wigginton B, Gartner C, Rowlands IJ (2017). Is it safe to vape? Analyzing online forums discussing e-cigarette use during pregnancy. Womens Health Issues.

[34] Sharma R, Wigginton B, Meurk C, Ford P, Gartner CE (2016). Motivations and limitations associated with vaping among people with mental illness: a qualitative analysis of Reddit discussions. Int J Environ Res Public Health.

[35] Li Q, Zhan Y, Wang L, Leischow SJ, Zeng DD (2016). Analysis of symptoms and their potential associations with e-liquids' components: a social media study. BMC Public Health.

[36] Hua M, Alfi M, Talbot P (2013). Health-related effects reported by electronic cigarette users in online forums. J Med Internet Res.

[37] Chen AT, Zhu S, Conway M (2015). What online communities can tell us about electronic cigarettes and hookah use: a study using text mining and visualization techniques. J Med Internet Res.

[38] Lee AS, Hart JL, Sears CG, Walker KL, Siu A, Smith C (2017). A picture is worth a thousand words: electronic cigarette content on Instagram and Pinterest. Tob Prev Cessat.

[39] Collins KM, Onwuegbuzie AJ, Jiao QG (2007). A mixed methods investigation of mixed methods sampling designs in social and health science research. J Mix Methods Res.

[40] Thompson KS, Davis G, Frankfort-Nachmias C (2007). Study Guide to Accompany Research Methods in the Social Sciences, Chava Frankfort-Nachmias, David Nachmias. Seventh Edition.

[41] Adkison SE, O'Connor RJ, Bansal-Travers M, Hyland A, Borland R, Yong H, Cummings KM, McNeill A, Thrasher JF, Hammond D, Fong GT (2013). Electronic nicotine delivery systems: international tobacco control four-country survey. Am J Prev Med.

[42] Dockrell M, Morrison R, Bauld L, McNeill A (2013). E-cigarettes: prevalence and attitudes in Great Britain. Nicotine Tob Res.

[43] Etter J, Bullen C (2011). Electronic cigarette: users profile, utilization, satisfaction and perceived efficacy. Addiction.

[44] Pepper JK, Brewer NT (2014). Electronic nicotine delivery system (electronic cigarette) awareness, use, reactions and beliefs: a systematic review. Tob Control.

[45] Duke JC, Lee YO, Kim AE, Watson KA, Arnold KY, Nonnemaker JM, Porter L (2014). Exposure to electronic cigarette television advertisements among youth and young adults. Pediatrics.

[46] Krishnan-Sarin S, Morean ME, Camenga DR, Cavallo DA, Kong G (2015). E-cigarette use among high school and middle school adolescents in Connecticut. Nicotine Tob Res.

[47] Kim M, Popova L, Halpern-Felsher B, Ling PM (2019). Effects of e-cigarette advertisements on adolescents' perceptions of cigarettes. Health Commun.

[48] Cummins SE, Zhu S, Tedeschi GJ, Gamst AC, Myers MG (2014). Use of e-cigarettes by individuals with mental health conditions. Tob Control.

[49] de Andrade M, Hastings G, Angus K, Dixon D, Purves R (2013). Cancer Research UK.

[50] Majeed BA, Weaver SR, Gregory KR, Whitney CF, Slovic P, Pechacek TF, Eriksen MP (2017). Changing perceptions of harm of e-cigarettes among US adults, 2012-2015. Am J Prev Med.

