# Supplementary material for: Perceptions and Sentiments About Electronic Cigarettes on Social Media Platforms: Systematic Review
Source: JMIR Public Health Surveill. 2020 Jan 15;6(1):e13673. doi: 10.2196/13673 (PMC6996744; doi:10.2196/13673)
Supplement: Multimedia Appendix 1 [file publichealth_v6i1e13673_app1.docx]

Table 1. Overview of included articles.

| First author, year | Study design | Social media platforms/online forums | Sampling | | Methods (data collection and analysis) | Phenomena or purpose of interest | Relevant discussion |
| --- | --- | --- | --- | --- | --- | --- | --- |
| Allem, 2017 [1] | Qualitative | Twitter | Stratified sampling | | Data collection using GNIP^a^ (Twitter API^b^); data analysis: content analysis, chi-square test | Track the popularity of the “Still Blowing Smoke” (anti-e-cig^c^ campaign) and “Not Blowing Smoke” (pro-e-cig campaign) over time on Twitter, and analyze the content of the messages, and determined those involved in these discussions | Pro-e-cig campaign (California Department of Public Health) was more referenced than anti-e-cig Twitter campaign with tweets against government regulation of e-cig refuting claims that e-cig manufactures were aligned with big tobacco and touting the health benefits of e-cig use. |
| Ayers, 2017 [2] | Quantitative and qualitative | Twitter | Purposive sampling | | Data collection using Twitter API; data analysis: content analysis, chi-square test | Identify reasons for using ENDS^d^ longitudinally from postings on Twitter | Findings from 2012 reveal that reason for using the e-cig was quitting combustible (the most cited reason); social image; able to use indoor; choices of flavor; and safety relative to combustibles on Twitter. On the other hand, by 2015, social image (most cited reason), quitting combustibles, and favorable odor being top three reasons for use. |
| Burke-Garcia, 2017 [3] | Quantitative and qualitative | Twitter | Purposive sampling | | Data collection using GNIP (Twitter API) and Radian6; data analysis: context and valence (sentiment) analysis, descriptive statistics | Compare two tools for data mining the content of social media using e-cig tweets to better understand the implications of e-cig content in social media | Themes identified were similar among the two data tools analyzing e-cig-related tweets: feasibility, poster type (individual/organization), context (tweet content analysis), and valence (positive/negative). However, there were some differences in cost and ease of use of the tools. |
| Chu, 2017 [4] | Quantitative and qualitative | Instagram | Stratified random sampling | | Data collection using Instagram API and *MySQL* database queries; data analysis: image analysis, Kruskal-Wallis tests, Dunn tests | Investigate vaping and e-cig-related images on Instagram | Overall, advertisement-themed images were most frequently displayed on Instagram, followed by product, activity, and then text among e-cig-related postings. Activity-themed and product-themed images appeared to have more *likes* and comments compared with advertisement-themed images. |
| Glowacki, 2017 [5] | Quantitative and qualitative | Twitter | Purposive sampling | | Data collection using *MDigitalLife Health Ecosystem* database; data analysis: text mining analysis | Assess how physicians from the United States and the United Kingdom talk about e-cigs on Twitter and identify the e-cig topics perceived to be salient | While the most common topic in the US tweets were related to dangerous e-cig use among teens displaying negative sentiment toward e-cig, UK tweets, on the other hand, had no mention of youth e-cig use and emphasized e-cig should be promoted widely because it can serve as an effective aid for smoking cessation. |
| Lee, 2017  [6] | Quantitative | Instagram, Pinterest | Stratified random sampling | | Data collection using Iconosquare; data analysis: content analysis, descriptive statistics | Examine e-cig content in visual materials posted on the social media platforms: Instagram and Pinterest | Both Instagram and Pinterest revealed that the majority of postings were posted with marketing intentions. Two-thirds of users on Pinterest shared information about customizing e-cig products such as modifying e-cig devices for functional aesthetic purposes, and with marketing purposes on Instagram. |
| Sharma, 2017 [7] | Qualitative | Reddit | Purposive sampling | | Data manually collected; data analysis: thematic analysis | Understand the nature and significance of online lay discussions about e-cigs and mental illness | Themes that motivated the e-cig use (eg, cost, health, or as an alternative to psychotropic medications) were also framed as reasons to not use them. Reasons for the interest in e-cig were identified as an aid for smoking cessation, a form of self-medication, and as a hobby. |
| Wigginton, 2017 [8] | Qualitative | Reddit, UK Vapers, AAEC^e^, E-cigarette forum, Aussie Vapers, Baby Gaga, Vaping Underground, What to Expect, Momtastic (pregnancy forum), Totally Wicked E-Liquid, Baby Centre (United Kingdom), Baby Center (general) | Purposive sampling | | Data manually collected; data analysis: thematic discourse analysis | Analyze 13 online forums that discuss vaping during pregnancy, with an intent to explore how the posts debated the safety of e-cig use during pregnancy | Three common ways people spoke about the safety aspects of vaping during pregnancy using discursive strategies were identified: quitting (nicotine) cold turkey is not safe, vaping is yet better than smoking, and vaping is not worth the risk for babies. |
| Zhan, 2017 [9] | Qualitative | Reddit, JuiceDB^f^, Twitter | Purposive sampling | | Data collection using JuiceDB API, manual selection for Reddit and Twitter; data analysis: NLP^g^, LDA^h^ | Gain a systematic understanding of the characteristics of various types of social media, to provide deep insights into how consumers and policy makers effectively use social media to track e-cig-related content and adjust their decisions and policies | The topic distribution between *Reddit* and *JuiceDB* were significantly different (*P*<.001), which indicated that the user discussions focused on different perspectives across the platforms. Various topics for each dataset were found. *Reddit* included topics about purchasing e-cig products; flavor-related experiences and sentiments; discussion of e-liquid components; the relationship between e-cig and traditional tobacco products; and personal experience and questions. *JuiceDB* revealed about the throat hit^i^ and vapor production; flavors; product promotion and recommendations; and vaping experience. *Twitter* data were specified to campaigns debating e-cig ban regulations and petitions of social media campaign. |
| Dai, 2016 [10] | Quantitative and qualitative | Twitter | Purposive sampling | | Data collection using Twitter API; data analysis: content and text mining analysis | Seek general public attitudes regarding e-cigs using social media data | Mixed opinions were present among organic tweets about e-cigs (neutral 19.4%, anti 17.7%, and pro 10.8%). When viewing opinion polarities by country, Canada had the highest percentages of commercial tweets (61.6%) and Malaysia had the lowest (27.8%). The United States (39.7%), the United Kingdom (39.0%), and Hungary (50.4%) had the highest number of organic tweets, with the United Kingdom having the highest rate of support for e-cig (21.2%). Within the United States, top three states with the highest rates of tweets opposing use of e-cig were Arkansas (51.3%), Kentucky (45.1%), and Utah (43.5%), while those with the lowest rates of tweets opposing e-cig use were Wyoming (11.8%), Delaware (25.3%), and Vermont (25.4%). |
| Laestadius, 2016 [11] | Qualitative | Instagram | Purposive sampling | | Data collection using Iconosquare; data analysis: content analysis, descriptive statistics, F-statistics, Fisher exact tests | Analyze e-cig content to highlight public health challenges created by this content and support understanding of e-cig promotion and usage | Overall, corporate users (vendors, brands, and representatives) made up 58.5% of posts, whereas personal users (individuals and enthusiasts) made up 41.2%. There were no statistically significant differences in the presence of health and cessation content between the corporate and personal posts. There were no posts that were critical of e-cigs. |
| Lazard, 2016 [12] | Qualitative | Twitter | Purposive sampling | | Data collection using Twitter API and NUVI software; data analysis: text mining analysis, descriptive statistics | Detect and analyze prevalent themes among the public conversation surrounding new regulations set by the FDA^j^ in May 2016 that deem e-cig as a part of regulatory tobacco product. Understand initial reactions of the public, and how this participation in the current discussion fits within the current regulatory landscape | The study revealed many expressed comments, opinions, words and phrases commonly associated with advocating for vaping and support for use of e-cig. |
| Li, 2016 [13] | Qualitative | Reddit | Purposive sampling | | Data manually collected; data analysis: content (symptom) analysis, descriptive statistics | Aim to mine the potential relationships between symptoms and e-liquid components, such as PG^k^, VG^l^, flavor extracts, and nicotine, using user-generated data | The majority of users reported negative sentiments about symptoms, including cough and respiratory systems problems/issues. Throat (n=150) and mouth (n=50) symptoms were the most commonly reported symptoms, and they tended to have positive sentiment. This is probably because the most direct perception of feeling after vaping an e-cig was in the throat and mouth. Many users enjoyed a slight throat hit to simulate the feeling of smoking traditional cigarettes. However, there still existed a large group of users who did not like throat hit. |
| Kavuluru, 2016 [14] | Quantitative | Twitter | Random sampling | | Data collection using Twitter API; data analysis: sentiment and thematic analysis, text mining and classification model; logistic regression | Identify e-cig proponents on Twitter via a high accuracy supervised predictive model and analyze the quantitative variation of tweeting behavior along popular themes when compared with other Twitter users | Despite the small percentage of e-cig proponents on Twitter who wrote about e-cig, the proponents tweeted more often (2-5 times) compared with other users and were 10 times more likely than others to highlight favorable, but not often scientifically corroborated, aspects of e-cig use. |
| Unger, 2016 [15] | Quantitative and qualitative | Twitter | Purposive sampling | | Data collection using Twitter API and NCapture program; data analysis: content analysis, chi-square test | Identify and analyze the content of tweets related to secondhand e-cig aerosol | Overall, coding by sentiment was depicted as neutral (neutral 39.2%, pro 34.9%, and anti 25.8%). Topic was predetermined as either health, social, advertisement, or unknown where every topic could categorize into pro, anti, or neutral. Among the topics, social tweets significantly outnumbered health tweets. Social tweets predominantly portrayed as pro-e-cig, and the health-related tweets as anti. |
| van der Tempel, 2016 [16] | Quantitative and qualitative | Twitter | Purposive sampling | | Data collection using *Sysomos HeartBeat*; data analysis: content and thematic analysis; authority-based analysis; descriptive statistics, chi-square tests | Examine message sources, themes, and attitudes within Twitter communications about e-cigs and smoking cessation | Among sample on Twitter, attitudes about e-cigs such as smoking cessation aids were favorable across user types (industry, press, public figures, fake accounts, and personal users), except for public health professionals, who lacked consensus and contributed negligibly to the conversation. The most common theme was the claim that e-cigs were more economical and efficient nicotine delivery systems than the conventional cigarette smoking. |
| Chen, 2015 [17] | Quantitative | Vapor Talk, Hookah Forum, Reddit | Purposive sampling | | Data collection using Wget, a Web crawler; data analysis: text mining (topic modeling), heat map visualization | Examine content from the websites: Vapor Talk, Hookah Forum, and Reddit to understand people’s experiences with different tobacco products; identify contextual factors of tobacco use behaviors, such as setting, time, social relationships, and sensory experience | E-cigs provided a different experience of e-cig use as compared with combustible cigarettes. Much of the discussion focused on symptoms that people were experiencing while using e-cigs. People using e-cigs appeared less likely to engage in the psychological battle of quitting by having e-cig divert their attention to a different activity, dealing with concrete problems to avert particular physiological symptoms associated with e-cig use. |
| Chu, 2015 [18] | Quantitative | GLOBALink | Purposive sampling | | Data collection using *MySQL* database; data analysis: social network and content analysis, descriptive statistics, independent *t* test, linear regression | Examine how e-cigs are addressed in *GLOBALink*, an online international tobacco control community, and understand the characteristics of sentiment toward e-cigs in discussion topics between countries with different network characteristics | Despite the growth of e-cig message postings, the overall trend in sentiments did not noticeably become more positive or negative. Although sentiment regarding e-cigs was generally more negative than other topics discussed in *GLOBALink*. The network analysis found a core/periphery structure where central countries focused on active positive discussion pertaining to e-cigs, whereas isolated and peripheral countries posted negative topics without many responses. |
| Cole-Lewis, 2015 [19] | Quantitative and qualitative | Twitter | Random sampling | | Data collection using GNIP (Twitter API); data analysis: content analysis; descriptive statistics, correlation analyses | Identify key conversation trends and patterns over time using historical Twitter data about e-cig use | Tweets were positive in sentiment (>92%) and frequently posted by everyday people (49.6%) followed by the e-cig community movement (29.3%). Retailors accounted for the majority of tweets (19.7%) containing images, and marketing-related tweets were twice as likely to contain an image (17.3%) compared with the average rate at which images occur in tweets (8.3%). Everyday people represented 62.3% of news tweets as compared with reputable news sources that accounted for 6.4% of news and 0.96% of information tweets. Foundations/organizations provided 3.98% of information tweets and 5.2% of news tweets. Also, 32.4% of marketing tweets came from everyday people compared with 26.2% from retailors and 6.4% from tobacco companies. |
| Harris, 2014 [20] | Quantitative and qualitative | Twitter | Purposive sampling | Data collection using Node XL; data analysis: content analysis, descriptive statistics, network visualization | | Examine the messages and tweet patterns in social media response to the Chicago Department of Public Health e-cig campaign | Less than 10% were in support of e-cig regulation (propolicy), whereas 89.2% were opposed (antipolicy). Overall, propolicy tweets were most likely to focus on regulation (8.6%) and science (3.7%), whereas antipolicy tweets were most likely to focus on safety (58.8%) and lies/propaganda (36.8%). More than half of antipolicy tweets were about use of e-cigs for cessation as a healthier alternative to combustible cigarettes (58.8%). Tweet sentiment was significantly associated with location (*P*<.001) indicating Chicago residents were significantly more likely expected to send a propolicy tweet than other residents. |
| Hua, 2013 [21] | Quantitative | Electronic cigarette forum; Vapers forum; Vapor Talk | Purposive sampling | Data manually collected; data analysis: content analysis | | Document the positive and negative short-term health effects produced by e-cig use through an analysis of original posts from three online e-cig forums | A total of 405 different symptoms because of e-cig use were reported from three forums. Of the reported effects, 81.9% were negative, 17.8% were positive, and less than 0.1% were neutral. All systems had both positive and negative symptoms, except for urogenital, which had only negative effects. Negative symptoms were often described as persistent, worsened, or increasing. In contrast, positive effects were often described as decreased, improved, or eliminated. While the reported health effects were similar in all three forums, the forum with the most posts was analyzed in detail. Effects, which were reported for 12 organ systems/anatomical regions, occurred most often in the mouth and throat and in the respiratory, neurological, sensory, and digestive systems. Users with negative symptoms often reported more than one symptom, and in these cases, interactions were often seen between systems, such as the circulatory and neurological systems. Positive effects usually occurred singly and most frequently affected the respiratory system. |

^a^GNIP: social media API aggregation company.

^b^API: application program interface.

^c^e-cig: electronic cigarette(s).

^d^ENDS: electronic nicotine delivery system

^f^AAEC: All About E-Cigarettes.

^e^DB: database.

^g^NLP: natural language processing.

^h^LDA: latent Dirichlet allocation, a generative model for topic modeling Lexicon-based extraction approach.

^i^throat hit: feeling of smoke hitting the back of the throat.

^j^FDA: Food and Drug Administration.

^k^PG: propylene glycol.

^l^VG: vegetable glycerin.

References

1. Allem JP, Escobedo P, Chu KH, Soto DW, Cruz TB, Unger JB. Campaigns and counter campaigns: reactions on Twitter to e-cigarette education. Tob Control 2017;26(2):226-229. doi: [10.1136/tobaccocontrol-2015-052757](https://doi.org/10.1136/tobaccocontrol-2015-052757). PMID: 26956467
2. Ayers JW, Leas EC, Allem JP, Benton A, Dredze M, Althouse BM, Cruz TB, Unger JB. Why do people use electronic nicotine delivery systems (electronic cigarettes)? A content analysis of Twitter, 2012-2015. PLoS One 2017;12(3):e0170702. doi: [10.1371/journal.pone.0170702](https://doi.org/10.1371/journal.pone.0170702). PMID: 28248987
3. Burke-Garcia A, Stanton CA. A tale of two tools: reliability and feasibility of social media measurement tools examining e-cigarette twitter mentions. Informatics in Medicine Unlocked 2017;8:8-12. [doi:10.1016/j.imu.2017.04.001](https://doi.org/10.1016/j.imu.2017.04.001).
4. Chu KH, Allem JP, Cruz TB, Unger JB. Vaping on Instagram: Cloud chasing, hand checks and product placement. Tob Control 2017;26(5):575-8. doi:[10.1136/tobaccocontrol-2016-053052](https://dx.doi.org/10.1136%2Ftobaccocontrol-2016-053052). PMID: [27660111](https://www.ncbi.nlm.nih.gov/pubmed/27660111)
5. Glowacki EM, Lazard AJ, Wilcox GB. E-cigarette topics shared by medical professionals: a comparison of tweets from the United States and United Kingdom. Cyberpsychol Behav Soc Netw 2017;20(2):133-137. doi: [10.1089/cyber.2016.0409](https://doi.org/10.1089/cyber.2016.0409). PMID: 28118024
6. Lee AS, Hart JL, Sears CG, Walker KL, Siu A, Smith C. A picture is worth a thousand words: Electronic cigarette content on Instagram and Pinterest. Tob Prev Cessat 2017;3. doi: [10.18332/tpc/74709](https://dx.doi.org/10.18332%2Ftpc%2F74709). PMID: [28815224](https://www.ncbi.nlm.nih.gov/pubmed/28815224)
7. Sharma R, Wigginton B, Meurk C, Ford P, Gartner CE. Motivations and limitations associated with vaping among people with mental illness: a qualitative analysis of Reddit discussions. Int J Environ Res Public Health 2016;14(1):pii E7. doi: [10.3390/ijerph14010007](https://doi.org/10.3390/ijerph14010007). PMID: 28025516
8. Wigginton B, Gartner C, Rowlands IJ. Is it safe to vape? Analyzing online forums discussing e-cigarette use during pregnancy. Womens Health Issues 2017;27(1):93-9. [doi:10.1016/j.whi.2016.09.008](https://doi.org/10.1016/j.whi.2016.09.008). PMID: 27773530
9. Zhan Y, Liu R, Li Q, Leischow SJ, Zeng DD. Identifying topics for e-cigarette user-generated contents: a case study from multiple social media platforms. J Med Internet Res 2017;19(1). doi: [10.2196/jmir.5780](https://doi.org/10.2196/jmir.5780). PMID: 28108428
10. Dai H, Hao J. Mining social media data for opinion polarities about electronic cigarettes. Tob Control. 2017;26(2):175-80. doi: [10.1136/tobaccocontrol-2015-052818](https://doi.org/10.1136/tobaccocontrol-2015-052818). PMID: 26980151
11. Laestadius LI, Wahl MM, Cho YI. # Vapelife: an exploratory study of electronic cigarette use and promotion on Instagram. Subst Use Misuse 2016;51(12):1669-1673. doi: [10.1080/10826084.2016.1188958](https://doi.org/10.1080/10826084.2016.1188958). PMID: 27484191
12. Lazard AJ, Wilcox GB, Tuttle HM, Glowacki EM, Pikowski J. Public reactions to e-cigarette regulations on Twitter: A text mining analysis. Tob Control 2017;26(e2):e112-6. [doi: 10.1136/tobaccocontrol-2016-053295](http://dx.doi.org/10.1136/tobaccocontrol-2016-053295). PMID: 28341768
13. Li Q, Zhan Y, Wang L, Leischow SJ, Zeng DD. Analysis of symptoms and their potential associations with e-liquids’ components: a social media study. BMC Public Health 2016;16(1):674. doi: [10.1186/s12889-016-3326-0](https://doi.org/10.1186/s12889-016-3326-0). PMID: 27475060
14. Kavuluru R, Sabbir AK. Toward automated e-cigarette surveillance: spotting e-cigarette proponents on Twitter. J Biomed Inform 2016;61:19-26. doi: [10.1016/j.jbi.2016.03.006](https://doi.org/10.1016/j.jbi.2016.03.006). PMID: 26975599
15. Unger JB, Escobedo P, Allem JP, Soto DW, Chu KH, Cruz T. Perceptions of secondhand e-cigarette aerosol among Twitter users. Tob Regul Sci 2016;2(2):146-52. doi: [10.18001/TRS.2.2.5](https://dx.doi.org/10.18001%2FTRS.2.2.5). PMID: [28090560](https://www.ncbi.nlm.nih.gov/pubmed/28090560)
16. van der Tempel J, Noormohamed A, Schwartz R, Norman C, Malas M, Zawertailo L. Vape, quit, tweet? Electronic cigarettes and smoking cessation on Twitter. Int J Public Health 2016;61(2):249-256. doi: [10.1007/s00038-016-0791-2](https://doi.org/10.1007/s00038-016-0791-2). PMID: 26841895
17. Chen AT, Zhu SH, Conway M. What online communities can tell us about electronic cigarettes and hookah use: a study using text mining and visualization techniques. J Med Internet Res 2015;17(9):e220. doi: [10.2196/jmir.4517](https://doi.org/10.2196/jmir.4517). PMID: 26420469
18. Chu KH, Valente TW. How different countries addressed the sudden growth of e-cigarettes in an online tobacco control community. BMJ Open 2015;5(5):e007654. doi: [10.1136/bmjopen-2015-007654](https://doi.org/10.1136/bmjopen-2015-007654). PMID: 25998038
19. Cole-Lewis H, Pugatch J, Sanders A, Varghese A, Posada S, Yun C, Schwarz M, Augustson E. Social listening: a content analysis of e-cigarette discussions on Twitter. J Med Internet Res 2015;17(10). doi: [10.2196/jmir.4969](https://doi.org/10.2196/jmir.4969). PMID: 26508089
20. Harris JK, Moreland-Russell S, Choucair B, Mansour R, Staub M, Simmons K. Tweeting for and against public health policy: response to the Chicago Department of Public Health's electronic cigarette Twitter campaign. J Med Internet Res 2014;16(10):e238. doi: [10.2196/jmir.3622](https://doi.org/10.2196/jmir.3622). PMID: 25320863
21. Hua M, Alfi M, Talbot P. Health-related effects reported by electronic cigarette users in online forums. J Med Internet Res 2013;15(4):e59. doi: [10.2196/jmir.2324](https://doi.org/10.2196/jmir.2324). PMID: 23567935
