# Supplementary material for: Perceptions and Sentiments About Electronic Cigarettes on Social Media Platforms: Systematic Review
Source: JMIR Public Health Surveill. 2020 Jan 15;6(1):e13673. doi: 10.2196/13673 (PMC6996744; doi:10.2196/13673)
Supplement: Multimedia Appendix 2 [file publichealth_v6i1e13673_app2.docx]

Table 2. Sentiments and themes identified.

| First author, year | Search queries (keywords used to search) | Number of content/post analyzed | Data collection period | Social media platform | Overall sentiment of discussion toward e-cig^a^ use (Pro, Anti, Neutral, Mixed, or N/A) | Identified themes of summarized message topics | Example of health-related comments or posts |
| --- | --- | --- | --- | --- | --- | --- | --- |
| Allem, 2017 [1] | #stillblowingsmoke OR stillblowingsmoke OR still blowing smoke OR stillblngsmoke OR “still blng smok” OR #notblowingsmoke OR notblowingsmoke OR “not blng smoke” OR @CAPublicHealth | 2192 tweets | March to June 2015 | Twitter | *Pro* (Pro=92%, Neutral=6%, Anti=2%) | *Regulation/propaganda* (35%): perceived government overreach, perceived lies being spread by the government; *Health* (30%): cessation, scientific studies, the effects of vaping on health; *Money* (10%): taxes, small businesses, tobacco sales; *Marketing elements* (8%): specific products, coupons, and vape shops | “A new study verifies that e-cigarettes are orders of magnitude safer than tobacco cigarettes.” |
| Ayers, 2017 [2] | electronic cigarette(s), electronic cig(s), e cig(s), e-cig(s), eking(s), e cigarette(s), e-cigarette(s), ecigarette(s), vape(s), vaper(s), vaping | 2900 tweets each year | 2012 and 2015 | Twitter | *Pro* | *Reasons for using e-cig as reported on Twitter in 2012*: Quitting combustibles (43%); social image (21%); can vape indoors (17%); flavor choices (14%); safe to use (9%); low cost (3%); and favorable order (2%); *Reasons for using e-cig as reported on Twitter in 2015:* Social image (37%); quitting combustibles (29%); flavor choices (13%); can vape indoors (12%); favorable odor (5%); and low cost | “My ecig helped me quit smoking.” “I want one of those e-cigs, it will make me look cool!” |
| Burke-Garcia, 2017 [3] | “e-cigarettes”, “vaping”, “e-cigarettes health”, “vaping health” | 1000 tweets | August 2015 | Twitter | *Neutral* (Neutral=88-90%, Pro=6%, Anti=4%-5%) | *Radian6* Endorsement (29%); marketing/sales (18%); other (17%); product characteristics (12%); utilization patterns (12%); health/consequences (6%); policy (4%); cessation (1%); consumer purchases (1%); *GNIP*^b^ *(Twitter API*^c^*)* Endorsement (24%); marketing/sales (23%); product characteristic (21%); other (16%); policy (4%); utilization patterns (4%); health/consequences (3%); consumer purchases (1%); cessation (0%) | RT^d^ “@StopVaping: RETWEET this if your not VAPING today because you want to live” |
| Chu, 2016 [4] | #ecig, #ejuice, #eliquid, #vape, #vaping, #vapelife | 2208 posts | October to December 2015 | Instagram | *Pro* | *Advertisement* (29%, eg, a photo edited with embedded text of a company name, *Pro*); *Product* (28%, eg, a photo of an e-cig, vapor, or e-juice, *Pro*); *Other* (22%, images with no clear classification and spanned a variety of areas beside e-cigs, *Pro*); *Activity* (18%, eg, a person blowing vapor); *Text* (2%, eg, common “meme” or text-based poster, *Pro*). | None |
| Glowacki, 2017 [5] | Vaping, #Vaping, VAPE, #Vape, ecig, #Ecig, E-cigarette, #Ecigarette, E-cig, electronic cig, electronic-cig | 3800 tweets | June 2015 to June 2016 | Twitter | *Mixed* (The United States: Anti=54%, Pro=28%, Neutral: 18%; The United Kingdom: Pro=43%, Anti=37%, Neutral=19%) | *Topics identified from US physician-generated tweets:* Likelihood of e-cig use among teenagers and e-cigs as a gateway to tobacco use (21%); FDA^e^ regulations (18%); the potential for e-cigs to help cigarette smokers quit (16%); effects of flavoring chemicals on the lungs (12%); England’s favorable recommendation on e-cigs (12%); e-cig advertising aimed at teenagers (11%); and support for raising the required age for purchasing e-cigs (10%); *Topics identified from UK physician-generated tweets:* Debate among users about the pros and cons of e-cigs compared with those of traditional cigarettes (19%); the need to promote e-cigs as an aid for smoking cessation (18%); need for measures of the source of harm (18%); the benefits of having general practitioners encourage their smoking patients to switch to e-cigs (13%); references to a Harvard study on the effects of flavoring chemicals on the lungs (12%); Public Health England’s recommendation that e-cigs are safer than traditional forms of tobacco use (12%); and complications that e-cigs can cause for individuals who have had breast reconstruction surgery (7%) | None |
| Lee, 2017 [6] | vapelife, vapor, vapelyfe, vapeporn, ecig, vaping, ecigs, electroniccigarette | Instagram=900, Pinterest=900 | Period not mentioned (5 weeks) | Instagram, Pinterest | *Pro* | *Marketing* (53.1%, *Pro*): sampling, products, information about retailors and manufacturers; *Customization* (50.8%, *Pro*): functional and aesthetic purposes of e-cig devices; *Juice or flavors* (9.72%, *Pro*): popular and new flavors, ideas on creating novel flavors; *Featured professional or amateur models using e-cigs* (8.22%, *Pro*): seductive or provocative images, products, enticing use, garnering attention; *Performing tricks* (7.8%, *Pro*): exhaling vapor in creative ways (eg, producing vapor rings or blowing vapor out of mouth and sucking it back into the nose); *Meme* (7.67%, *Neutral*); *E-cigs as a replacement for traditional cigarettes and asserted that e-cig use is better* (4.83%, *Pro*): potential health benefits, economic and environmental savings; *Celebrities* (3.11%, *Pro*): “hip” and “cool,” legitimizing use; *Health benefits* (2.66%, *Pro*): personal experiences, manufacturer claims; *Marijuana use in e-cig devices* (2.0%, *Pro*): sharing updated technology for achieving the best “high” or experiences; *Social acceptance* (1.33%, *Pro*): convenience of smoking even in restricted areas as supposed to cigarettes | N/A^f^ |
| Sharma, 2017 [7] | electronic cigarettes, e-cigarettes, vaping AND psychiatric, mental illness, schizophrenia, depression, psychosis, anxiety, bipolar | 3263 comments (from 133 discussion threads) | April 2016 | Reddit | N/A | *Motivations for using e-cigs with people with mental illness:* Self-medication (symptom relief); quitting smoking; freedom and control (feeling); hobby (symptom relief); social connectedness; and encouragement from caregivers; *Limitations or barriers to using e-cigs:* Unsatisfactory substitute for cigarettes and psychiatric medicines; drug interactions; nicotine addiction; risks of electronic liquid; practical difficulties; and cost | “I vape, no side effects except better concentration and appetite reduction-which counteracts the lithium.” |
| Wigginton, 2017 [8] | “vaping during pregnancy” | 448 posts in 13 online forums | April 2012 to October 2018 | Reddit, UK Vapers, AAEC^g^, E-cigarette forum, Aussie Vapers, Baby Gaga, Vaping Underground, What to Expect, Momtastic (pregnancy forum), Totally Wicked E-Liquid, Baby Centre (The United K), Baby Center (general) | N/A | The safety of e-cig during pregnancy: *Quitting (nicotine) cold turkey is unsafe:* symptoms of nicotine withdrawal (eg, stress, high blood pressure, anxiety); *Vaping is the lesser of two evils:* harm reduction approach; *Vaping is not worth the risk:* unknown risks associated with vaping, no scientific evidence to support vaping during pregnancy and for the baby | “I am vaping right now. There are some things to take into consideration concerning quitting and vaping: Like some other mommas here, I have anxiety issues, and also told by my doctor that quitting cold turkey could risk making my blood pressure go out of whack and even put the fetus in distress.” |
| Zhan, 2017 [9] | e cigarettes, electronic cigarettes, ecigarettes, ecigs, smoking electronic cigarettes, smoking ecigarettes, and smoking ecigs, E-cig ban, e-cigarette ban | (a) Reddit=27,638, (b) JuiceDB^h^=14,433, (c) Twitter=13,356 | (a) January 2011 to June 2015, (b) June 2013 to November 2015, (c) January 2010 to June 2015 | Reddit, JuiceDB, Twitter | *Pro* (Reddit: Pro=60.7% opponents on e-cig bans; Neutral=29.9%; Anti=9.4% proponents on e-cig bans) | Comprehensive topics identified across platforms: *Promotions:* Sales from vendors to users and trades among users (*Reddit*, 10%), which shows pattern of the large secondhand e-cig trading market, raising new concerns in regulations and surveillance; production, promotion, and recommendations on JuiceDB; *Flavor discussions:* Fairly consistent themes of flavors were identified (*Reddit*: fruits, cream, tobacco, menthol, beverages, sweet, seasonings, and nuts; *JuiceDB*: sweet, fruity, rich, creamy, spiced, tobacco, cool, nutty, and coffee); *Experience sharing* (feeling and features): Vaping methods and features such as steeping, throat hit^i^, and vapor production were discussed on *Reddit* and on *JuiceDB*; *Regulation debates:* *Reddit* provided negative attitude (60.7%) toward regulations using reasons of personal freedom and experience, safer product compared with traditional tobacco products, politics driven, appealed for actions to down bills designed to ban e-cigs. *Twitter* was used to launch campaigns using certain hashtags (eg, no harm, smoking cessation and saving lives, pharma interests/tax income, biased research, personal freedom and rights, simple opposition, call to action, only tag, and neutral descriptions). No policy-related discussion on *JuiceDB*. | None |
| Dai, 2016 [10] | e-cig, e-cigarette, e-liquid, vape, vaping, vapor, vaporizer | 757,167 tweets | July to October 2015 | Twitter | *Neutral* (Neutral**=**19.4; Anti=17.7%; Pro=10.8%) | *Content against (anti) tweets:* educational and advocating content about potential risks of e-cigs to stop vaping (17.7%); *Socioeconomic factors:* higher education rates more likely to tweet about e-cigs; higher percentage of black and African-American population associated with higher prevalence rate of organic e-cigarette tweets; higher median household income associated with higher prevalence rate of e-cig organic tweets; states within the United States with a higher percentage of persons under 18 years old associated with lower rates of support to e-cigs; states with a higher population percentage of females associated with higher rates of tweets in support of e-cigs. | None |
| Laestadius, 2016 [11] | #ecig, #vape | 85 posts (43 #ecig, 43 #vape) | October 2014 to October 2015 | Instagram | *Neutral* (Neutral=81.2%; Pro=48.3%) | *Health promotion and smoking cessation* *(Pro: 48.3%)*: e-cigs as cessation devices (23.5%), e-cigs healthier than tobacco cigarettes (16.5%), e-cig as “healthy” (7.1%), e-cigs environmentally friendly (1.2%), e-cigs cheaper than tobacco cigarettes (0%); *Use and depiction of e-cigs (Neutral)*: electronic juice content (62.4%), mention of mods or building (60.0%), e-cig device in image, not in use (36.5%), e-cig part in image (25.9%), e-cig being used in image (15.3%); *Shared identity/community (Neutral):* Presence of identity: 81.2% | None |
| Lazard, 2016 [12] | “electronic cigarettes” FDA, “electronic cigarette” FDA, “e-cig” FDA, ecig FDA, ecigs FDA, #ecgs FDA, “electronic cigarettes” #FDA, “electronic cigarette” #FDA, “e-cig”, #FDA, ecig #FDA, ecigs #FDA, #ecig #FDA, “electronic cigarettes” Rules, “electronic cigarette” Rules, “e-cig” Rules, ecig Rules, ecigs Rules, #ecig Rules, “electronic cigarettes” Ruling, “electronic cigarette” Ruling, “e-cig” Ruling, ecig Ruling, ecigs Ruling, #ecig Ruling, “electronic cigarettes” regulation, “electronic cigarette” regulation, “e-cig” regulation, ecig regulation, ecgis regulation, #ecig regulation, “electronic cigarettes” regulations, “electronic cigarette” regulation, “electronic cigarettes” regulations, “electronic cigarette” regulations, “e-cig” regulations, ecig regulations, ecigs regulations, #ecig regulations | 4629 tweets | May 2016 | Twitter | *Pro* (Pro=68%, Neutral=32%, Anti=0%) | E-cig industry is threatened by the FDA’s new regulations (34.3%, *Pro*); Ban to sell to minors (13.7%, *Neutral*); Challenges against FDA deeming and concerns for vaping consumers moving forward (11.4%, *Pro*); Questioning the role and stance of politicians for the negative impact of the policy (9.8%, *Neutral*); How FDA deeming will benefit Big Tobacco (8.2%, *Pro*); News story highlighting negative impact of the regulation on e-cig manufacturers (4.6%, *Pro*); Press article regarding the main e-cig regulations including FDA deeming (4.5%, *Neutral*); YouTube video created by vaping advocate discussing deeming regulations (4.4%, *Pro*); Mixed review of the government’s new “oversight” of e-cig (1.5%, *Mixed*) | None |
| Li, 2016 [13] | electronic cigarettes, e-cigarettes, ecigarettes, e-cigs, flavor, flavors, e-juice | 3605 posts | January 2011 to June 2015 | Reddit | N/A | *Negative sentiment of symptoms* (referred to as sadness, aversion, uncomfortableness, pain, etc): respiratory (lung, cough, and phlegm), neurological (headache, sleep disorder, and fatigue), allergy and cavity, digestive, sensory (hearing), chest (pain and tightness); *Positive sentiment of symptoms* (referred to as happiness, praise, affection, enjoyment, etc): Sinus, neurological (tingle and focus), mouth and throat | “Before I even finished my first fill-up, I noticed a tightness in my upper lungs while exhaling, and an urge to cough that far exceeds the typical dripper’s throat-clearing feeling...” |
| Kavuluru, 2016 [14] | electronic-cigarette, e-cig, e-cigarette, e-juice, e-liquid, vape-juice, and vape-liquid | 1000 Tweeter profiles | September to December 2013 and March 2015 | Twitter | *Pro,* proponents versus others: mean positive scores (0.92 and 0.79); mean negative scores (0.01 and 0.03) | *Flavors* (menthol, strawberry, blueberry, cola, cherry, and mint): Proponents were 15 times more likely to tweet about e-cig flavors (2013 sample) and are 20 times more likely to do (2015 sample); *Harm reduction* (reduced harm, less harmful, safer than, safer alternative, and healthy/healthier alternative): Proponents were 60 times more likely to tweet about this aspect. The ratio was increased 4.6 times from 2013 to 2015, compared with other users; *Smoke-free aspect* (smoke-free, smoke-less): Proponents were more likely to tweet about this aspect than others. The ratio was increased 3 times from 2013 to 2015; *Smoking cessation* (giving up, quitting, kicking smoking/tobacco): Proponents were more likely to tweet about this aspect than others. The ratio was increased 6 times from 2013 to 2015. However, the absolute volume of cessation-related tweets decreased given the 2015 data had over 100,000 more tweets than of 2013. | None |
| Unger, 2016 [15] | secondhand vape, secondhand vaping, second-hand vape, second-hand vaping, vape smoke, ecig smoke, e-cig smoke, e-cigarette smoke, vape shs, ecig shs, vape secondhand smoke, vape second-hand smoke, esmoke, e-smoke | 1,519 tweets | February to April 2015 | Twitter | *Neutral* (Neutral=39.24%; Pro=34.96%; Anti=25.81%) | *Social: Pro:* vape smoke tricks/performance-based video links, freedom and social benefits of vaping; *Anti:* blowing e-cig aerosol at other people is obnoxious or annoying, people who perform with vaping and their smoke on social media are juvenile or uncool; *Health: Pro:* the use of e-cigs to quit smoking, the relative safety of e-cigarette aerosol, or enjoyment of e-cig aerosol; *Anti:* short-term and long-term health effects of exposure to e-cig aerosol (eg, headache, eye irritation, nausea, and lung disease); *Neutral:* public’s need for more information); *Advertisement* | “Your secondhand e-cig smoke is giving me lung cancer.” |
| van der Tempel, 2016 [16] | ecigarette, vaping, #ecigs, #vapelife (filtered using terms quit and stop smoking) | 600 tweets | 2014 | Twitter | *Pro*= Attitude: Complete sample versus Industry-free sample (Pro=79% vs 62%; Anti=12% vs 17%; Neutral=8% vs 21%); Affective content: Complete sample versus Industry-free sample (Pro=46% vs 27%; Anti=7% vs 15%) | Themes identified in the messages: *Marketing* (34%); *News articles* (27%); *First-person experience* (17%: Pro: 86%, Neutral: 12%, and Con: 2%); *Neutral information* (12.9%); *Humor* (7.7%); *Just starting e-cigs* (5.2%); *Others:* advocating e-cigs; more economical than smoking; second-person experience; healthy and safe way to quit; personal opinion; attempt to engage other Twitter users; offering advice; nicotine craving; tastes good; using or comparing to other substance/NRTs^j^; policy-related; getting others started. | None |
| Chen, 2015 [17] | e-cigarette, vaping, hookah, health, forum, stopsmoking, electronic cigarette, hookah | Vapor Talk=13,814 posts; Hookah Forum=17,761 posts; Reddit=134,712 posts | April to June 2014 | Vapor Talk (forum “General E-cig Discussion” and “Health and Safety”); Hookah Forum; Reddit (forum “electronic_cigarette,” “hookah,” and “stopsmoking”) | N/A | E-Cig versus Combustible Cigarette Use: *Symptoms:* large portion of the discussion in the *Vapor Talk Health Safety* (ie, health dangers of smoking cigarettes, problems that forum members have encounter in the mouth and throat, the use of PG^k^ as opposed to VG^l^, and sleep quality); *Psychology:* most salient category (60.60%) in the *Stopsmoking* subreddit (ie, overcoming cravings, dealing with friends, encouragement that cravings would pass); *Quitting methods:* 15.29% in the *Stopsmoking* subreddit (ie, “cold turkey,” “gum,” and “patch”).  E-Cig versus Hookah: *Buying and selling equipment for e-cigarettes and hookah:* the consumers in *Vapor Talk* appeared to primarily be end consumers, whereas the consumers. There were also individuals in both forums (Vapor Talk, Hookah) whose member type indicated vendor; *Technique:* In *Vapor Talk*, topics concerning technique included how to get a good taste and how different characteristics of the juices affect the vaping experience. Overall, e-cig and hookah forums were similar in that their members were actively engaged in information exchange concerning technical and cost-related aspects of the use of their products of choice; *Health:* a substantive part of the conversation in *Vapor Talk* focused on vaping as opposed to smoking “analogs” (traditional cigarettes), and though not as prominent in the discussion content | None |
| Chu, 2015 [18] | e-cig, e cig, electronic-cig’ and electronic cig*quitting sm*, *quitsmok*, *cessation* | 853 posts | July 2005 to April 2012 | GLOBALink | *Pro* (Pro=61.9%, Anti=47.7%, Neutral=8.6%) | - Core countries that actively participated in the discussion threads: The United States, Australia, Canada, Switzerland, and the United Kingdom. - Posts generating from central countries in the network (ie, Canada, Switzerland, the United States, Australia, and the United Kingdom) discussed in a positive manner, whereas isolated and peripheral countries (ie, Pakistan, Japan, Israel, Malaysia, etc) posted negative topics without much interaction and responses. - Top 12 thread summarized message topic were about asking for information, health information, general information, industry packaging, country bands of e-cig. | None |
| Cole-Lewis, 2015 [19] | Vaping, Vape, Vaper, Vapers, Vapin, Vaped, Evape, Vaporing, e-cig*a, ecig*a, e-pen, epen, e-juice, ejuice, e-liquid, eliquid, cloud chasing, cloudchasing, deeming AND regulation, deeming AND FDA, deemed AND FDA, Deem*a and FDA (*a= word stem used to identify any word beginning with these characters) | 10,128 | May 2013 to April, 2014 | Twitter | *Mixed* (Changing trend from Pro to Anti to Neutral; initially, Pro=71.11%, Neutral=16.78%, Anti=12.11%, but showed steady decline in positive sentiment from December 2013) | Tweet distribution by theme: *Advertisement/promotion* (26.29%); *Policy/government* (20.16%), *Health and safety* (13.10%); *Cessation* (6.3%); *Flavors* (4.45%); *Underage usage* (4.18%); *Craving* (3.89%); Tweet distribution by user description: Everyday person (64.99%), e-cigarette community movement (15.92%), retailor (7.77%), bot/hacked (6.9%), tobacco company (1.97%), foundations/organization (1.2%), reputable news source (0.72%), celebrity (0.44%), government (0.08%); Tweet distribution by genre: Personal opinion (28.14%), marketing (21.15%), first person e-cig use or intent (20.30%), information (14.41%), news/update (8.18%), and second/third person experience (7.87%) | None |
| Harris, 2014 [20] | #ECigs, #ECigTruths, #kcavo, #vaping @ChiPublicHealth | 683 tweets | January 2014 | Twitter | *Pro* (Pro=89.2% opponents of e-cig regulation (antipolicy); Anti=7.5% proponents of e-cig regulation (propolicy); Neutral=3.4% unable to tell) | *Safety:* e-cigs are harmful, foster nicotine addiction, promote smoking (propolicy=2.0%) versus e-cigs are safer than alternative, promote cessation (antipolicy=52.4%); *Lies/propaganda:* propaganda/lie spread by e-cig industry or supporter (propolicy=0%) versus propaganda. Lie spread by health department or other government (antipolicy=32.8%); *Health effect:* studies find some ingredients are carcinogenic, increased use by kids; need more research (propolicy=2.8%) versus science shows e-cigs contain only nicotine and water, no dangerous secondhand vapor (antipolicy=31.9%); *Flavors:* sweet flavors are for kids (propolicy=0.3%) versus adults like flavors too (antipolicy=3.7%); *Regulation:* ingredients, look, and use are like cigarettes, should be regulated like cigarettes (propolicy=6.4%) versus regulation is a slippery slope, do not need nanny state (antipolicy=24.7%); *Need for attention:* e-cigs are an important threat to public health (propolicy=0%) versus health department should focus on more serious health threats (antipolicy=2.3%) | @ChiPublicHealth it’s not about being safe, it’s about being SAFER than the alternative #EcigsSaveLives it’s about HARM REDUCTION #Casaa |
| Hua, 2013 [21] | electronic cigarette forum (on *Google* search engine) | Electronic Cigarette Forum posts =543; Vapers Forum posts =34; Vapor Talk posts =55 | July 2011 | Electronic cigarette forum; Vapers forum; Vapor Talk | *Anti,* Anti=80.5% (negative symptoms), Pro=19.3% (positive symptoms); Neutral=0.02% | Symptoms category: *Negative:* included “swelling” and “dehydration”; *Positive:* included “improved stamina” and “improved overall health.”  Sample of 8 health-related effects reported by e-cig users grouped by positive and negative according to the organ system/anatomical region: *Respiratory system* (n=74): Positive=asthma relief, improved sinuses, eliminating morning cough, eliminating snoring, improved COPD^m^, improved sleep apnea; Negative=sinus infection, dry cough, nasal infection, choking (after use), discomfort in lungs, shortness of breath; *Mouth and throat* (n=68): Positive=improved sore throat, improved itchy throat, decrease dental plaque, eliminated morning breath; Negative=thrust, dry lips, burning throat, burning throat, sore lips; *Neurological system* (n=52): Positive=sleep better, eliminating depression, improved nerve pain, decrease seizures, positive mood changes; Negative=vivid dreams, insomnia, cold sweats, mood changes, hypersomnia, depression; *Sensory system* (n=39): Positive=improved night vision, eliminates glaucoma and eye bulges, improved sense of taste, improved visual acuity, improved smell; Negative=metallic mouth taste, eye pain, irrigated eyes, distorted vision, dry eyes, eye redness; *Digestive system* (n=36): Positive=controlled appetite, eliminates stomach pain, eliminates acid reflex, improved upset stomach, improved bowel movement; Negative=increased cramps, increased heartburns, weight loss and gain, ingestion, upset stomach; *Muscular/skeletal system* (n=29): Positive=decreased muscle jerks, eliminates/decreased fibromyalgia pain; Negative=backache, stiff/sore hand muscles, head/neck aches, hand strain, join pain; *Integumentary* (n=29): Positive=improved skin, improved eczema, eliminated facial rash, improved dry skin; Negative=acne, oily skin, itchy skin, rash with burning sensation, hands stained (electronic juice) | None |

^a^e-cig(s): electronic cigarette(s).

^b^GNIP: social media API aggregation company.

^c^API: application program interface.

^d^RT: retweet.

^e^FDA: Food and Drug Administration.

^f^N/A: not applicable.

^g^AAEC: All About E-Cigarettes.

^h^DB: database.

^i^throat hit: feeling of smoke hitting the back of the throat.

^j^NRT: nicotine replacement therapy.

^k^PG: propylene glycol.

^l^VG: vegetable glycerin.

^m^COPD: chronic obstructive pulmonary disease.

References

1. Allem JP, Escobedo P, Chu KH, Soto DW, Cruz TB, Unger JB. Campaigns and counter campaigns: reactions on Twitter to e-cigarette education. Tob Control 2017;26(2):226-229. doi: [10.1136/tobaccocontrol-2015-052757](https://doi.org/10.1136/tobaccocontrol-2015-052757). PMID: 26956467
2. Ayers JW, Leas EC, Allem JP, Benton A, Dredze M, Althouse BM, Cruz TB, Unger JB. Why do people use electronic nicotine delivery systems (electronic cigarettes)? A content analysis of Twitter, 2012-2015. PLoS One 2017;12(3):e0170702. doi: [10.1371/journal.pone.0170702](https://doi.org/10.1371/journal.pone.0170702). PMID: 28248987
3. Burke-Garcia A, Stanton CA. A tale of two tools: reliability and feasibility of social media measurement tools examining e-cigarette twitter mentions. Informatics in Medicine Unlocked 2017;8:8-12. [doi:10.1016/j.imu.2017.04.001](https://doi.org/10.1016/j.imu.2017.04.001).
4. Chu KH, Allem JP, Cruz TB, Unger JB. Vaping on Instagram: Cloud chasing, hand checks and product placement. Tob Control 2017;26(5):575-8. doi:[10.1136/tobaccocontrol-2016-053052](https://dx.doi.org/10.1136%2Ftobaccocontrol-2016-053052). PMID: [27660111](https://www.ncbi.nlm.nih.gov/pubmed/27660111)
5. Glowacki EM, Lazard AJ, Wilcox GB. E-cigarette topics shared by medical professionals: a comparison of tweets from the United States and United Kingdom. Cyberpsychol Behav Soc Netw 2017;20(2):133-137. doi: [10.1089/cyber.2016.0409](https://doi.org/10.1089/cyber.2016.0409). PMID: 28118024
6. Lee AS, Hart JL, Sears CG, Walker KL, Siu A, Smith C. A picture is worth a thousand words: Electronic cigarette content on Instagram and Pinterest. Tob Prev Cessat 2017;3. doi: [10.18332/tpc/74709](https://dx.doi.org/10.18332%2Ftpc%2F74709). PMID: [28815224](https://www.ncbi.nlm.nih.gov/pubmed/28815224)
7. Sharma R, Wigginton B, Meurk C, Ford P, Gartner CE. Motivations and limitations associated with vaping among people with mental illness: a qualitative analysis of Reddit discussions. Int J Environ Res Public Health 2016;14(1):pii E7. doi: [10.3390/ijerph14010007](https://doi.org/10.3390/ijerph14010007). PMID: 28025516
8. Wigginton B, Gartner C, Rowlands IJ. Is it safe to vape? Analyzing online forums discussing e-cigarette use during pregnancy. Womens Health Issues 2017;27(1):93-9. [doi:10.1016/j.whi.2016.09.008](https://doi.org/10.1016/j.whi.2016.09.008). PMID: 27773530
9. Zhan Y, Liu R, Li Q, Leischow SJ, Zeng DD. Identifying topics for e-cigarette user-generated contents: a case study from multiple social media platforms. J Med Internet Res 2017;19(1). doi: [10.2196/jmir.5780](https://doi.org/10.2196/jmir.5780). PMID: 28108428
10. Dai H, Hao J. Mining social media data for opinion polarities about electronic cigarettes. Tob Control. 2017;26(2):175-80. doi: [10.1136/tobaccocontrol-2015-052818](https://doi.org/10.1136/tobaccocontrol-2015-052818). PMID: 26980151
11. Laestadius LI, Wahl MM, Cho YI. # Vapelife: an exploratory study of electronic cigarette use and promotion on Instagram. Subst Use Misuse 2016;51(12):1669-1673. doi: [10.1080/10826084.2016.1188958](https://doi.org/10.1080/10826084.2016.1188958). PMID: 27484191
12. Lazard AJ, Wilcox GB, Tuttle HM, Glowacki EM, Pikowski J. Public reactions to e-cigarette regulations on Twitter: A text mining analysis. Tob Control 2017;26(e2):e112-6. [doi: 10.1136/tobaccocontrol-2016-053295](http://dx.doi.org/10.1136/tobaccocontrol-2016-053295). PMID: 28341768
13. Li Q, Zhan Y, Wang L, Leischow SJ, Zeng DD. Analysis of symptoms and their potential associations with e-liquids’ components: a social media study. BMC Public Health 2016;16(1):674. doi: [10.1186/s12889-016-3326-0](https://doi.org/10.1186/s12889-016-3326-0). PMID: 27475060
14. Kavuluru R, Sabbir AK. Toward automated e-cigarette surveillance: spotting e-cigarette proponents on Twitter. J Biomed Inform 2016;61:19-26. doi: [10.1016/j.jbi.2016.03.006](https://doi.org/10.1016/j.jbi.2016.03.006). PMID: 26975599
15. Unger JB, Escobedo P, Allem JP, Soto DW, Chu KH, Cruz T. Perceptions of secondhand e-cigarette aerosol among Twitter users. Tob Regul Sci 2016;2(2):146-52. doi: [10.18001/TRS.2.2.5](https://dx.doi.org/10.18001%2FTRS.2.2.5). PMID: [28090560](https://www.ncbi.nlm.nih.gov/pubmed/28090560)
16. van der Tempel J, Noormohamed A, Schwartz R, Norman C, Malas M, Zawertailo L. Vape, quit, tweet? Electronic cigarettes and smoking cessation on Twitter. Int J Public Health 2016;61(2):249-256. doi: [10.1007/s00038-016-0791-2](https://doi.org/10.1007/s00038-016-0791-2). PMID: 26841895
17. Chen AT, Zhu SH, Conway M. What online communities can tell us about electronic cigarettes and hookah use: a study using text mining and visualization techniques. J Med Internet Res 2015;17(9):e220. doi: [10.2196/jmir.4517](https://doi.org/10.2196/jmir.4517). PMID: 26420469
18. Chu KH, Valente TW. How different countries addressed the sudden growth of e-cigarettes in an online tobacco control community. BMJ Open 2015;5(5):e007654. doi: [10.1136/bmjopen-2015-007654](https://doi.org/10.1136/bmjopen-2015-007654). PMID: 25998038
19. Cole-Lewis H, Pugatch J, Sanders A, Varghese A, Posada S, Yun C, Schwarz M, Augustson E. Social listening: a content analysis of e-cigarette discussions on Twitter. J Med Internet Res 2015;17(10). doi: [10.2196/jmir.4969](https://doi.org/10.2196/jmir.4969). PMID: 26508089
20. Harris JK, Moreland-Russell S, Choucair B, Mansour R, Staub M, Simmons K. Tweeting for and against public health policy: response to the Chicago Department of Public Health's electronic cigarette Twitter campaign. J Med Internet Res 2014;16(10):e238. doi: [10.2196/jmir.3622](https://doi.org/10.2196/jmir.3622). PMID: 25320863
21. Hua M, Alfi M, Talbot P. Health-related effects reported by electronic cigarette users in online forums. J Med Internet Res 2013;15(4):e59. doi: [10.2196/jmir.2324](https://doi.org/10.2196/jmir.2324). PMID: 23567935
